# The Current State of Research in the Field of Photosensitizers and Photoactivators for Photodynamic/Photothermal Cancer Therapy: A Review

**DOI:** 10.3390/ijms262110733

**Published:** 2025-11-04

**Authors:** Pavel Yudaev, Yulia Aleksandrova, Elena Chugunova, Margarita Neganova

**Affiliations:** 1 Nesmeyanov Institute of Organoelement Compounds, Russian Academy of Sciences, Vavilova St., 28, Bld. 1, Moscow 119991, Russia; yulia.aleks.97@mail.ru; 2 Arbuzov Institute of Organic and Physical Chemistry, FRC Kazan Scientific Center, Russian Academy of Sciences, Kazan 420088, Russia; chugunova.e.a@gmail.com

**Keywords:** photodynamic therapy, complex, porphyrins, ligands, cancer, photosensitizer, photoactivator

## Abstract

This review is devoted to research in the field of photodynamic and photothermal therapies for malignant tumors. Special attention in the review is given to photosensitizers based on compounds with a tetrapyrrole ring system, their metal complexes, BODIPY and aza-BODIPY derivatives, squaraines, and photoactivators based on metal complexes with other ligands such as phenanthroline and its derivatives, metronidazole, pyridine, and imidazole derivatives. Additionally, the review considers nanosized carriers for photosensitizers, such as organic and inorganic nanoparticles, liposomes, and extracellular vesicles. This review also discusses the dark toxicity and phototoxicity of these compounds and the processes of free oxygen radical formation, mitochondrial dysfunction, and induction of apoptosis in cancer cells. It has been established that nanoscale delivery systems are more promising for use in photodynamic and photothermal therapy compared to molecular photosensitizers. This is due to their improved solubility in physiological environments, selective accumulation in tumors, prolonged photoactivity, and lower therapeutic dose, which allows for the minimization of the side effects of treatment. Among the molecular photosensitizers under consideration, amphiphilic tetrapyrroles appear to be the most promising. Specifically, tetrapyrrole complexes of indium (III) and iridium (III) with non-porphyrin ligands exhibit favorable photophysical and biological characteristics. The review also indicates that photosensitizers tend to localize in the mitochondria of tumor cells, contributing to oxidative stress and apoptosis activation. This review may be of interest to biochemists and oncologists.

## 1. Introduction

According to the Global Cancer Observatory GLOBOCAN, cancer is one of the leading causes of death worldwide, accounting for 9.7 million deaths in 2022 [1]. Standard cancer treatments such as chemotherapy, radiation therapy, and surgery face a number of challenges. These include non-specific drug delivery to cancer cells, severe side effects [2], and an increased risk of complications for surgical methods [3]. Therefore, today, the search for new technologies for cancer therapy remains a relevant area of research.

Photodynamic therapy (PDT) is a promising alternative to these treatments for some oncological diseases, in particular in the treatment of inoperable solid tumors (sarcomas, gliomas, carcinomas) [4,5,6,7]. The advantages of PDT compared to surgical methods of cancer treatment include non-invasiveness, minimal functional impairment, and, compared to chemotherapy and radiation therapy, selectivity of therapeutic action, the ability to trigger antitumor immune responses [8], minimizing systemic toxicity, and preserving fertility [9].

**Figure 1:**
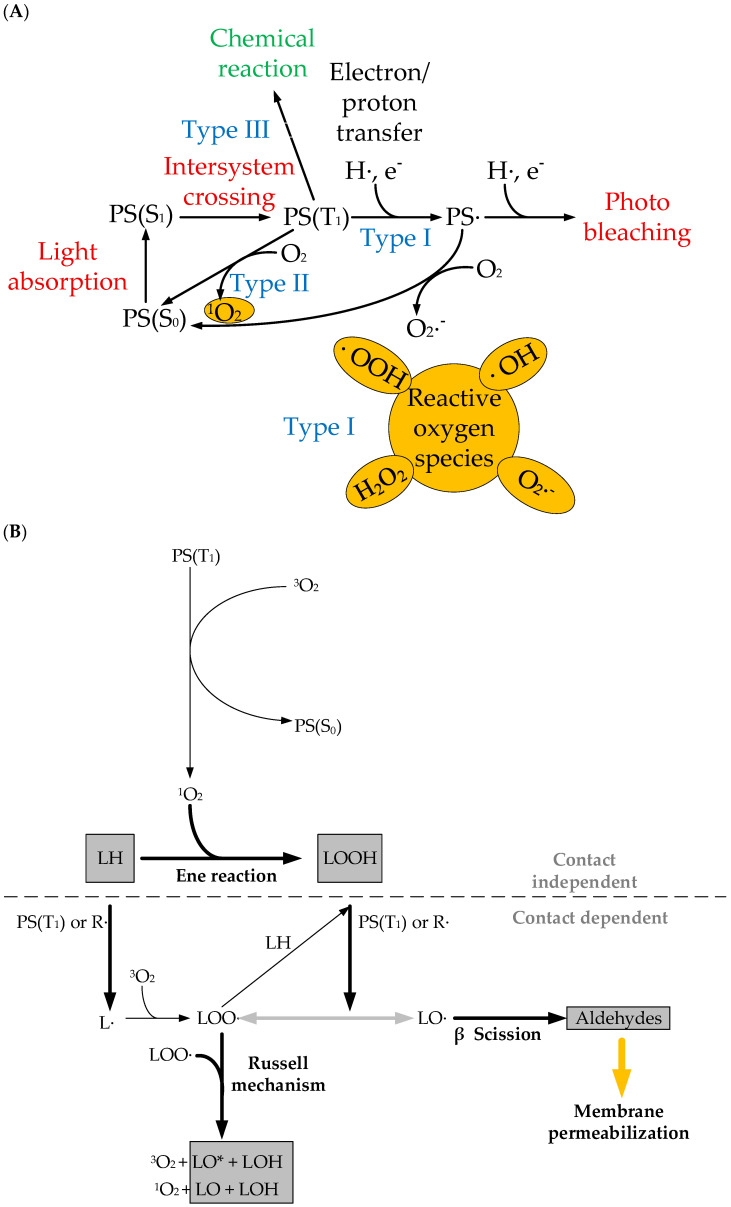
(**A**) Schematic diagram of the photosensitization mechanism. PS(S_0_) is the ground singlet state of PSs; PS(S_1_) is the excited singlet state; PS(T_1_) is the excited triplet state. Type I—PS^·^ reacts directly with a substrate (e.g., polyunsaturated fatty acids in cell membrane lipids) and transfers an electron or a proton, forming organic radicals. These radicals may further react with cellular oxygen to produce reactive oxygen species (ROS), such as a superoxide anion (O_2_^−^·), a hydroperoxide radical (HOO·), peroxides (H_2_O_2_, ROOH), and a hydroxyl radical (HO·), as well as initiate free radical chain reactions. Type II—PS^·^ can undergo triplet−triplet energy transfer to molecular oxygen (triplet in the ground state) to form excited-state singlet oxygen (^1^O_2_). Type III—oxygen-free photoactivation. (**B**) Chemical pathways for photoinduced membrane permeabilization. The map distinguishes between contact-independent and contact-dependent processes, which rely on ^1^O_2_ or on direct reactions between PSs and lipids, respectively. LH: nonoxidized lipid; L·, LOO·, LO·: lipid carbon-centered, peroxyl, and alkoxyl radicals; LOOH, LOH, LO, LO*: lipid hydroperoxide, alcohol, ketone, and excited-state ketone [14].

PDT is based on the photoactivation of photosensitizers (PSs) capable of absorbing light energy of a specific wavelength, transitioning from the ground state S_0_ to the excited singlet state S_1_ and then to the triplet excited state T1. This leads to the generation of reactive oxygen species (ROS) by two photochemical reactions of type I and II [10,11]. In type I reactions, PSs in the T1 state can react directly with biomolecules such as proteins or lipids, resulting in the formation of radicals that initiate free radical chain reactions. These reactions were discussed in detail in the review article [11]. On the other hand, in the type II reaction mechanism characteristic of most PSs, excitation energy from PSs in the T_1_ state is transferred to molecular oxygen (O_2_), resulting in the formation of singlet oxygen (^1^O_2_), which is extremely electrophilic and can cause damage to membranes, proteins, and DNA via cellular apoptosis or necrosis (Figure 1A) [12]. Mitochondrial membrane damage is followed by an increase in the Bax/Bcl-2 ratio, which leads to the subsequent activation of caspase-9. Once activated, caspase-9 cleaves and activates caspase-3 and -7, triggering the cleavage of poly[ADP-ribose] polymerase 1, PARP-1, and DNA fragmentation.

ROS generation via the type II photochemical reaction pathway is easier than that via type I, and most PSs used today for antitumor PDT are thought to operate via this mechanism. Both reaction types can occur simultaneously and compete with each other, depending on the PS type, tissue oxygen concentration, and pH of the environment [13].

Phototoxic effects of photosensitizers after light activation can also occur without the involvement of oxygen (mechanism type III). In oxygen-independent photodynamic therapy, energy transfer from the excited photosensitizer (in this case called a photoactivator) to target cells occurs through a chemical reaction. Specifically, type III includes oxygen-independent photochemistry, ferroptosis (oxidation in the presence of iron ions), and mechanisms based on autophagy.

Furthermore, protein and membrane damage are of particular importance for PDT and are necessary for the cytotoxic effect. Figure 1B summarizes the main steps of photoinduced membrane damage. The first step typically involves an “ene” reaction between the lipid LH and the singlet oxygen ^1^O_2_, the product of which is hydroperoxide LOOH. Irreversible damage occurs when a hydrogen atom is abstracted from the higher-fatty-acid LH, which has double bonds, resulting in the formation of an alkyl radical L. Molecular oxygen then attaches to the alkyl radical, resulting in the formation of the peroxyl radical LOO·, which, by reacting with another fatty acid LH, is capable of initiating a new oxidation cycle, leading to the formation of the unstable lipid hydroperoxide LOOH and another alkyl radical L. The continuation phase of the chain reaction involves the breakdown of lipid hydroperoxides into other intermediate radicals and the initiation of a new oxidation chain by the peroxyl radical LOO·. Under the influence of light, alkoxyl radicals LO· are formed between the triplet PS(T_1_) and the double bond of the lipid hydroperoxide, leading to chain termination. This process leads to the formation of truncated aldehydes and other products that permeabilize membranes [14].

The PDT method provides a selective therapeutic effect, sparing the surrounding normal tissues due to the preferential accumulation of the photosensitizer in the tumor tissue and limiting the laser irradiation to the target tissue. If necessary, PDT can be repeated several times and combined with classical methods of treating malignant neoplasms [15]. In addition, photodynamic therapy can be combined with photothermal therapy, in which photothermal molecules or nanoparticles (in particular, gold nanoparticles and graphene oxide nanoparticles) generate heat under the action of a laser and destroy tumor tissue by local hyperthermia [16,17,18,19,20].

Regardless of the PDT mechanism pathway, one of the necessary conditions for the formation of cytotoxic particles during this process is the efficient generation of a relatively long-lived triplet T_1_ state by the photosensitizer. It is possible to increase the efficiency of its generation and, consequently, the efficiency of its further transformation via pathways I and II, mainly as a result of the correct choice of the PS type, as well as due to its chemical modification—the formation of a coordinated (metal complex) or uncoordinated form, the introduction of “heavy” atoms (halogens) into the molecule, etc. [21].

**Figure 2:**
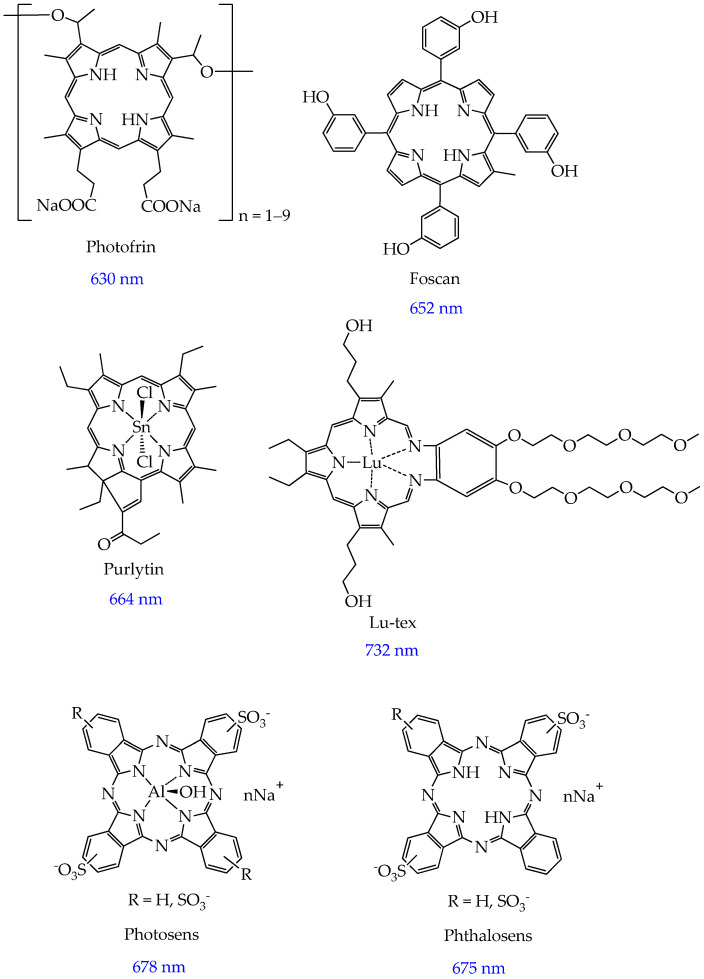
Structures of clinically tested photosensitizers.

The first photosensitizer to be approved by the FDA in 1993 for the photodynamic therapy of lung, bladder, and esophageal cancer was Photofrin [22]. Photofrin is a mixture of oligomeric derivatives of hematoporphyrin (Figure 2). However, despite the ease of obtaining Photofrin and its pronounced antitumor effect, it showed insufficient selectivity for cancer cells, accumulation in high concentrations in organs rich in reticuloendothelial elements (kidneys, spleen, liver) [23], and long-term (weeks) photosensitivity of the skin after PDT [24,25]. In addition, Photofrin has a weak absorption band in the red light spectrum (its activation wavelength is approximately 630 nm). Despite the fact that its absorption wavelength is in the therapeutic window (600–800 nm), Photofrin is not suitable for the treatment of deep-seated tumors [26].

An ideal photosensitizer should have, among other things, a high intersystem crossing efficiency to fill the T_1_ state, high accumulation in tumor cells, low toxicity in the dark, and a strong absorption at wavelengths in the therapeutic window. The shortcomings of Photofrin have forced researchers around the world to create compounds with improved properties, related to the second generation, which are characterized by selective accumulation in tumor cells without affecting healthy cells and improved characteristics of spectral lines with an absorption maximum of 650 nm and more, high quantum yields of singlet oxygen. Also, the use of second-generation PSs is characterized by a shorter duration of photosensitivity requiring light avoidance after drug administration [11].

Among the second-generation photosensitizers, Foscan (Germany), Purlytin (USA), and Lu-tex (USA) should be highlighted. Foscan or reduced porphyrin 5,10,15,20-tetra(m-hydroxyphenyl)chlorin is the first second-generation photosensitizer that has achieved clinical application in the treatment of squamous cell carcinoma of the head and neck at advanced stages (approved by the EMA in 2001 [5]). Compared to Photofrin, Foscan is an individual compound and has a maximum absorption at a wavelength of 652 nm, which facilitates deeper penetration of light into tumor tissue [27]. However, the disadvantages of Foscan include cases of tracheal and bronchial stenosis, esophagotracheal fistulas, and esophageal perforations observed in patients [5,28].

Purlytin is a tin ethyl etiopurpurin that is photoactivated at 664 nm. The longer activation wavelength of this PS provides an improved penetration depth (below 1 cm) [29] compared to Photofrin. Purlytin is FDA-approved for the treatment of metastatic breast cancer and Kaposi’s sarcoma. The disadvantage of Purlytin is prolonged (over a month) photosensitivity and dark toxicity [22,30].

Lu-tex or lutetium texaphyrin is a water-soluble macrocycle with a centrally coordinated Lu atom with a photoactivation wavelength of 732 nm [31]. A distinctive property of the photosensitizer Lu-tex is its high selectivity of accumulation in tumors (for example, in breast cancer cells), while, unlike Purlytin, Lu-tex is characterized by rapid elimination from the body [32]. Lu-tex is indicated for the treatment of prostate and breast cancer [14,22].

Among the second-generation non-porphyrin photosensitizers, modified phthalocyanines should be noted. These are aromatic heterocycles consisting of four isoindole rings connected by nitrogen atoms. Thus, in 1994, the second-generation photosensitizer Photosens was developed and underwent clinical trials at the Moscow Scientific and Production Association NIOPIK (Moscow). It is a water-soluble mixture of aluminum sulfophthalocyanines with an average degree of sulfonation of 3.4 (a mixture of di-, tri-, and tetra-substituted fractions) (Figure 2). Serious difficulties arising when working with phthalocyanines due to their high hydrophobicity were avoided by using sulfonated metal complexes [33]. Photosens is highly active and intensively absorbs light in the red region of the spectrum with a maximum in an aqueous solution at a wavelength of 678 nm. However, the disadvantage of Photosens, despite its hydrophilicity, is its slow cellular uptake. Phthalosens, a metal-free analogue of Photosens with a sulfo group number of 2.5, has a faster penetration into cells (Figure 2) [33].

Today, despite several photosensitizers being approved for clinical use, researchers are still searching for an ideal photosensitizer with the following properties: low dark and high light activity in therapeutic doses; high selectivity for accumulation in malignant tumor tissues and rapid elimination from normal tissues; strong absorption in the spectral range where biological tissues have the highest transmittance (red and near-IR ranges, 600–800 nm); excitation that does not overlap with absorption bands of endogenous substances such as melanin, hemoglobin, etc.; high quantum yield of singlet oxygen formation; low fluorescence quantum yield; ease of production or synthesis with uniform chemical composition; good solubility in water or liquids approved for intravenous administration; stability during storage; and favorable ADME properties (absorption, distribution, metabolism, and elimination) [34,35,36].

Additionally, the quantum yield and lifetime of singlet oxygen measured in organic solvents can decrease by an order of magnitude when moving to aqueous buffers due to PS aggregation, decreased O_2_ solubility, and a number of other reasons, not to mention biological fluids and tissues, where singlet oxygen ^1^O_2_ is quenched by various biomolecules [37].

To improve the penetration of second-generation photosensitizers through the lipid bilayer of cancer cells, improve solubility in biological environments, prevent their aggregation, minimize side effects, enhance ROS generation, and increase the accuracy and efficiency of targeting in target areas of the tumor and the destruction of neoplastic cells, a promising area of research is the development of nanoscale delivery systems for photosensitizers using their encapsulation or conjugation [38,39]. Such systems are usually referred to as third-generation photosensitizers. To date, metal nanoparticles have been studied as nanosized systems, for example, gold nanoparticles, silicon dioxide and titanium dioxide (IV) nanoparticles, oligomeric and polymeric nanoparticles, liposomes, and micelles [40,41].

Promising PSs as agents for conjugation or encapsulation are PSs obtained from natural components, such as chlorophyll compounds, which allow penetration to deeper levels in the body due to their similar structure to hemoglobin [42]. Thus, such drugs can circulate in the bloodstream almost anywhere. They do not need deeper penetration of light: tumor cells are simultaneously affected throughout the body, as a result of which, with the help of targeted delivery, it is possible to affect both the primary tumor and all secondary cancer types.

Over the past three years, review articles have been published on the use of photodynamic therapy for non-surgical treatment of various diseases (periodontitis [43], intraoral halitosis [44], psoriasis [45], cervical cancer [46]), for wound healing [47]. Other reviews cover the advantages of PDT over other cancer treatment methods, the mechanisms of action of PSs [48], the use of a combination of PDT with chemotherapy, immunotherapy, and gene therapy for cancer [36], and photoactive phytocompounds (furanocoumarins, alkaloids, curcuminoids, flavonoids, anthraquinones) [49], as well as methods to increase the efficiency of light energy capture by porphyrin photosensitizers [50]. However, to date, there are no review articles devoted to the comparative analysis of photosensitizers of different classes and nanosized carriers for photodynamic therapy of cancer. This review examines preclinical studies of the cyto- and phytotoxicity of molecular photosensitizers based on tetrapyrroles, metal complexes of tetrapyrroles, BODIPY derivatives, squaraines, nanosized carriers of photosensitizers, and photoactivators based on metal complexes.

## 2. Photosensitizers Based on Porphyrin Derivatives and Metal Complexes

Porphyrins are compounds with 18 π-electron planar macrocycles [51]. All porphyrin compounds are based on a conjugated macrocyclic ring consisting of tetrapyrrole residues linked together by methine bridges.

Research interest in porphyrins and their derivatives for use in photodynamic therapy remains high, as evidenced by the total number of articles in 2024 and 2025 of more than 600 according to the Scopus database. Porphyrins have shown absorption in the red light region, which is useful for treating deep-seated tumors (up to 1 cm) in clinical PDT, as longer red light is known to be less scattered and absorbed by tumor tissues and penetrates deeper compared to shorter blue light [52]. In addition, the ability of porphyrins to generate ROS upon absorption of light determines their antimicrobial and antiviral properties with a low risk of resistance development [53,54]. However, common porphyrins (for example, 5,10,15,20-tetraphenylporphyrin) also tend to aggregate in physiological environments due to their hydrophobicity and π-π interactions, which leads to decreased singlet oxygen yields and poor photostability [55,56].

**Figure 3:**
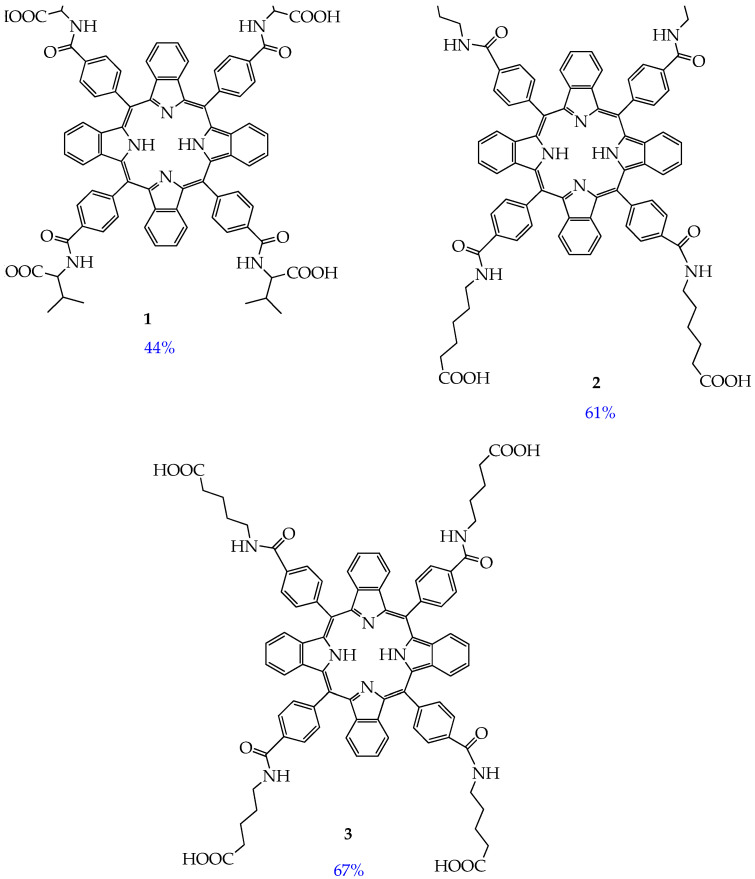
Structures of photosensitizers in preclinical trials [57]. The quantum yield of singlet oxygen is shown in blue. Quantum yield was measured using diphenylisobenzofuran as a scavenger and *meso*-tetraphenylporphyrin as the reference standard (Φ = 62%).

To eliminate the aggregation of common porphyrins, Jiang et al. proposed to use 5,10,15,20-tetra(4-substituted phenyl)tetrabenzoporphyrin derivatives (compounds **1–3**, Figure 3), which are π-expanded porphyrin derivatives with fused benzene rings, for photodynamic therapy [57]. The authors found that compounds **1–3** absorb light at a wavelength of approximately 650 nm without aggregation in water and normal saline (0.1% DMSO + 0.1% (*v*/*v*) polyoxyethylene castor oil). In our opinion, the absence of aggregation is due to the presence of hydrophilic carboxyl and amide groups in the structure of compounds **1–3**, which form hydrogen bonds with water.

**Figure 4:**
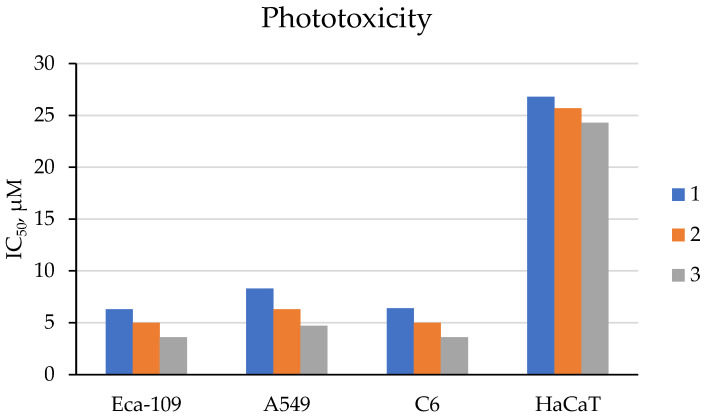
Photocytotoxicity of compounds **1–3** with 650 nm laser light at a dose of 12 J/cm^2^ [57].

Compounds **1–3** demonstrated high yields of reactive oxygen species (in the range of 0.44–0.67) after irradiation, with a predominance of the type II photosensitization mechanism (see Figure 1). The study of cytotoxicity of compounds **1–3** using the MTT assay on human esophageal squamous cell carcinoma Eca-109, human lung adenocarcinoma A549, and rat glioma C6 cells showed that compounds **1–3** exhibit low dark toxicity (IC_50_ > 30 μM). In turn, exposure to red light at a wavelength of 650 nm led to a sharp decrease in the IC_50_ value (IC_50_ less than 10 μM, Figure 4), indicating a potent photodynamic antitumor effect of compounds **1–3**. It was also found that the phototoxicity of compounds **1–3** towards immortalized human keratinocyte HaCaT cells is lower than that towards cancer cells (Figure 4), which indicates their selective effect on tumor cells.

In addition, the authors conducted an in vivo study involving Balb/c nude mice with Eca-109 tumors. Mice in the therapeutic groups were administered compound **3** with the best in vitro cytotoxicity at a dose of 2 mg/kg by injection into the tail vein followed by irradiation with a laser with a wavelength of 650 nm. The authors found that the tumor mass inhibition rate on day 10 was 94%. A study of the mechanism of antitumor action of compounds **1–3** showed that compounds **1–3** are localized predominantly in the mitochondria and lysosomes of the cancer cells rather than in their nuclei, and the main pathways of cancer cell death are mitochondrial membrane disruption, apoptosis, and necrosis.

In turn, amphiphilic porphyrins, which have a lipophilic part to facilitate passage through the bilayer of the cell membrane and mitochondrial membranes, as well as a hydrophilic part that ensures solubility in water and intravenous administration, usually have improved internalization and specific localization in tumor cells. These features have attracted much attention in studying their potential for PDT [58,59,60].

To improve solubility in water, anionic or cationic substituents and uncharged polar groups are usually introduced into the porphyrin core [58,61]. In addition, positively charged porphyrins have electrostatic interactions with negatively charged membranes and are therefore predominantly localized in the mitochondria [62]. A long alkyl chain can be added as a hydrophobic moiety to enhance cellular uptake of hydrophilic porphyrins. Besides higher and faster cellular uptake, tetracationic pyridine porphyrins with long alkyl chains showed suppressed lysosome localization and increased accumulation in organelles with negatively charged membranes such as mitochondria and endoplasmic reticulum (ER), which was not observed with hydrophilic analogues [63,64].

**Figure 5:**
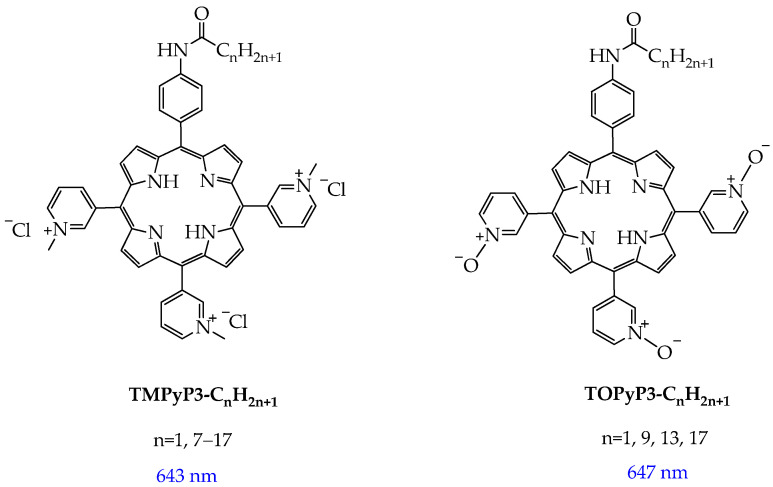
Structures of tetracationic pyridine porphyrins **TMPyP3-CH_3_, TMPyP3-C_n_H_2n+1_** n = 7–17, and **TOPyP3-C_n_H_2n+1_** (n = 9, 13, 17).

In [65], amphiphilic tetracationic pyridine porphyrins with a short **TMPyP3-CH_3_** and a long alkyl chain **TMPyP3-C_n_H_2n+1_** (n = 7–17) were synthesized (Figure 5).

**Figure 6:**
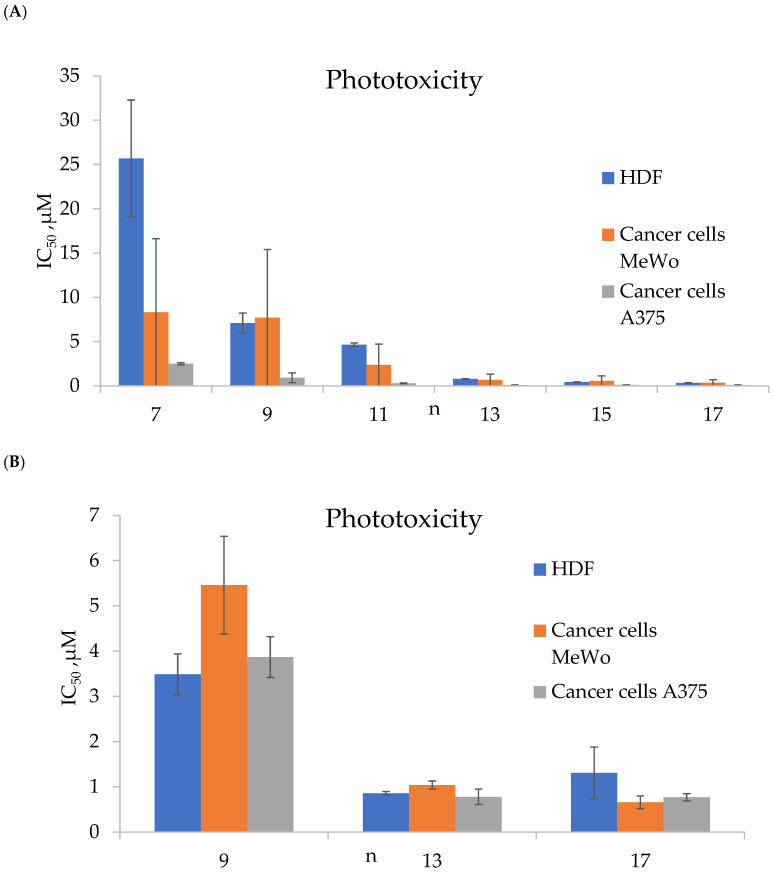
Phototoxicity of porphyrins **TMPyP3-C_n_H_2n+1_** (**A**) and **TOPyP3-C_n_H_2n+1_** (**B**) depending on the value of n (λ = 643 nm) [65,66].

For the synthesized porphyrins **TMPyP3-CH_3_** and **TMPyP3-C_n_H_2n+1_**, the authors established the following facts: (1) **TMPyP3-C_n_H_2n+1_** had high phototoxicity after irradiation with red light with a wavelength of 643 nm and selectivity towards melanoma cell lines (MeWo and A375) compared to human skin fibroblasts (HDFs) (Figure 6), while hydrophilic porphyrins **TMPyP3-CH_3_** had low phototoxicity (IC_50_ more than 100 μM); (2) with an increase in the length of the alkyl chain n, the phototoxicity of porphyrins **TMPyP3-C_n_H_2n+1_** improves, which is probably due to an increase in cellular uptake and the cellular absorption rate; (3) the amelanotic cell line (A375) was more sensitive to PDT than the melanotic MeWo, most likely due to the lack of melanin (Figure 6); (4) the long-alkyl-chain porphyrins **TMPyP3-C_n_H_2n+1_**, compared with the hydrophilic analogues **TMPyP3-CH_3_**, formed micelle-like vesicles in water, which could capture singlet oxygen ^1^O_2_ for a longer period; and (5) the longer-alkyl-chain porphyrins **TMPyP3-C_n_H_2n+1_** bound to albumin, probably through a hydrophobic pocket, and underwent caveolae-mediated endocytosis, while for the hydrophilic **TMPyP3-CH_3_**, transport across the membrane such as clathrin-mediated endocytosis was observed.

It has also been shown that amphiphilic porphyrins **TMPyP3-C_n_H_2n+1_** are predominantly localized in mitochondria and in the ER, which has a positive effect on the outcome of PDT.

The same research group synthesized not only N-methylated porphyrins but also their *N*-oxidized analogues **TOPyP3-C_n_H_2n+1_** (Figure 5). The authors found that **TOPyP3-C_n_H_2n+1_** are also effective for treating melanoma, but unlike *N*-methylated porphyrins, **TOPyP3-C_n_H_2n+1_** show a much smaller difference in selectivity between pigmented MeWo and non-pigmented A375 melanoma cells [66]. The IC_50_ values for human fibroblasts for *N*-oxidized analogues **TOPyP3-C_n_H_2n+1_** were greater than 100 μM, indicating their selectivity for cancer cells.

**Figure 7:**
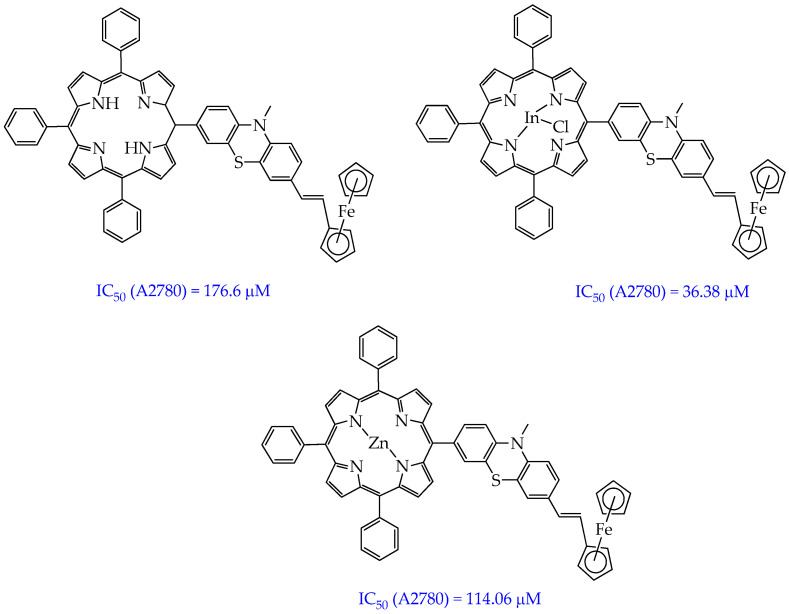
Structures of ferrocenylvinylphenothiazinylporphyrin and its metal complexes.

Porphyrin metal complexes with “high” metals often exhibit higher singlet oxygen quantum yields compared to free porphyrins. Phototoxicity of porphyrin derivatives can be modulated by the central metal ion. In particular, In(III) phenothiazinyl porphyrin complexes showed a higher singlet oxygen quantum yield (60%) compared to the free porphyrin precursor (12%) and zinc(II) complexes (29%) (Figure 7) [67]. In addition, In(III) ferrocenylvinylphenothiazinyl porphyrin (Figure 5) had higher phototoxicity towards A2780 ovarian cancer cells (IC_50_ 36.38 μM) compared to the free porphyrin (IC_50_ 176.6 μM) and zinc(II) complex (IC_50_ 114.06 μM). The higher quantum yield and phototoxicity are probably due to the influence of indium(III). “Heavy” paramagnetic indium(III), unlike “light” diamagnetic zinc(II), enhances the influence of the internal heavy atom on intersystem crossing, which ensures the generation of singlet oxygen.

The mechanism of antitumor action of the indium complex of porphyrin consisted of oxidative stress and a decrease in the secretion of nuclear factor erythroid 2-related factor 2 (Nrf-2) and an increase in the secretion of tumor necrosis factor TNF-α in A2780 tumor cells. The Nrf-2 factor is responsible for the protective antiapoptotic effect of A2780 tumor cells, and TNF-α, on the contrary, induces the apoptosis of A2780 tumor cells.

However, the compounds obtained in the work [67] absorb light with a lower wavelength (505 nm) than the previously considered amphiphilic porphyrins, which will lead to a lower depth of their penetration into tumor tissue.

**Figure 8:**
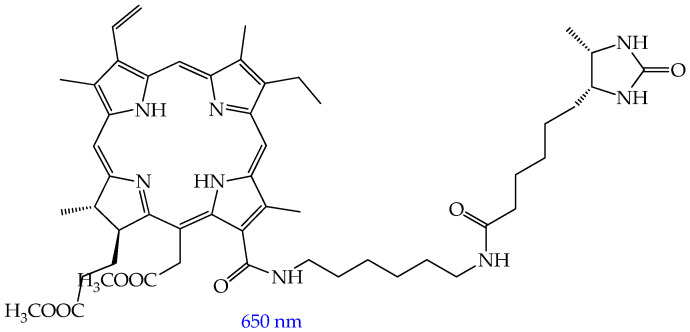
Structures of CDBTN.

Porphyrin derivatives and their metal complexes act as promising photosensitizers in combination with chemotherapeutic agents such as fluorouracil or *cis*-platinum. Rutkowski et al. [68] showed that therapy with the porphyrin derivative CDBTN (Figure 8) (50 nM) and fluorouracil (25 μM) was more effective (inhibition rate 80%) in terms of inhibition of triple-negative breast cancer BT-549 cells compared to CDBTN monotherapy (cell growth inhibition rate 34%) and CDBTN and *cis*-platinum therapy (41%).

The mechanisms of action of the photosensitizer and the chemotherapeutic agent include apoptosis, characterized by a decrease in the size of the nucleus, and autophagy, which is identified as the accumulation of cytosolic vacuoles and membranes. In this work, in vivo studies were performed using *Hydra viridissima*, an evolutionarily ancient animal with naturally occurring tumors characterized by differentiation, arrest, and uncontrolled accumulation of female germline progenitor cells. The authors found that only when using a combination of CDBTN and cis-platinum after irradiation of hydra with red light (650 nm wavelength, 4.8 J·cm^−2^ dose), the hydra produced a floral phenotype, implying that the photodynamic effect played a role in the changes. In contrast, the combination of CDBTN and fluorouracil produced the opposite effect, which the authors attributed to CDBTN protecting against fluorouracil. Thus, the in vivo results did not match the in vitro results on triple-negative breast cancer cells. The greatest harm to hydra from the combination of CDBTN and *cis*-platinum is likely due to the fact that hydra are more sensitive to the metallic platinum in *cis*-platinum than to other chemotherapeutic drugs.

**Figure 9:**
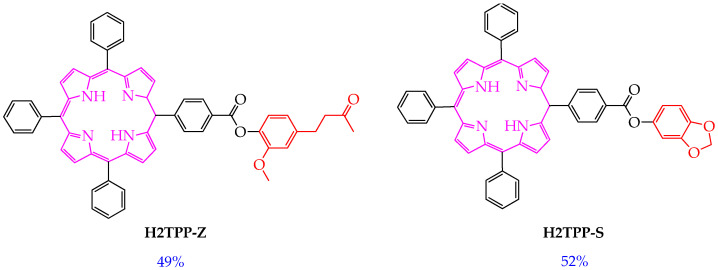
Structures of H2TPP-Z and H2TPP-S. To determine the quantum yield of singlet oxygen, 1,3-diphenylisobenzofuran in DMSO was used.

Also of interest to researchers are conjugates of porphyrin derivatives and compounds of natural origin. Such conjugation allows combining in one molecule the light-absorbing properties of the porphyrin fragment and the biological activity of low-molecular natural products. For example, in the work [69], the hybrid compounds **H2TPP-Z** and **H2TPP-S** were synthesized, including the structural core of tetraphenylporphyrin and subunits of zingerone and sesamol (Figure 9).

The authors showed that the synthesized compounds **H2TPP-Z** and **H2TPP-S** absorb light in the red light region (645 and 644 nm, respectively) and efficiently generate singlet oxygen with yields of 49% and 52%, respectively. The indicated values of singlet oxygen yields were higher than those of Foscan (30%). However, the antitumor effect of the compounds **H2TPP-Z** and **H2TPP-S** and the influence of the zingerone and sesamol subunits on the antitumor effect were not considered.

As mentioned above, the number of studies devoted to porphyrins for photodynamic therapy is in the several hundreds, so we decided to summarize the available data on their photophysical and biological properties in a table. Table 1 presents the photophysical and biological properties of some porphyrin derivatives and their metal complexes synthesized over the past two years. According to the presented data, a number of provisions can be highlighted: all the considered porphyrin derivatives and their metal complexes are soluble in water and DMSO and have high phototoxicity and no dark cytotoxicity; not all compounds were within the therapeutic window (600–800 nm) for photodynamic therapy, and most of them absorbed in the green and blue regions of the spectrum; the inclusion of D(+) glucose in porphyrin derivatives contributes to an increase in the quantum yield of ROS; and the main mechanism of antitumor action is apoptosis, not necrosis.

**Table 1:** Photophysical and biological properties of porphyrin derivatives and metal complexes (2024–2025).

Name	Λ Absorption, nm	Quantum Yield of Singlet Oxygen, %	Cell Line	Phototoxicity, μM	Dark Toxicity, IC50, μM	Mechanism	Ref.
5,10,15,20-tetrakis(N-ethylpyridinium-3-yl)porphyrin chloride (H_2_P)	520 (green light)	16.2	MDA- MB-231	0.21 ± 0.13	>1000	Apoptosis	[70]
Metallocomplex H_2_P and Sn(IV), SnP		17.3		0.77 ± 0.25	>1000		
AuP		<1		25.24 ± 12.11	>1000		
ZnP		41		0.22 ± 0.16	>1000		
Tetrakis (1-methylpyridinium-4-yl) p-toluenesulfonate porphyrin (TMPyP)	690	61	MDA- MB-231	60.1 ± 4.81	Cell viability above 90%	Apoptosis	[71]
			T47D	24.48 ± 1.99			
Sn (IV) complex of 5-(9-butyl-*9H*-carbazol-3-yl)-10,15,20-tris(4-(2-(2-methoxyethoxy)ethoxy)phenyl) porphyrin	433–610	43	A549	1.36	>50	Localization in ER, probably autophagy and apoptosis	[72]
Sn (IV) complex of *N*,*N*-diphenyl-4-(10,15,20-tris(4-(2-(2-methoxyethoxy)ethoxy)phenyl)porphyrin-5-yl)aniline	430–612	17		0.76			
In (III) complex of D(+) glucose-substituted tetrakis-(4- -ethylthiophenyl) porphyrin	415	63	MDA- MB-231	Cell viability 55.2%	Cell viability 100%	Not researched	[73]
Ga (III) complex of D(+) glucose-substituted tetrakis-(4- -ethylthiophenyl) porphyrin		63		Cell viability 50.7%			

Thus, the most promising compounds for use in the field of photodynamic therapy of tumors are amphiphilic porphyrins, their metal complexes with heavy paramagnetic metals, and synergistic mixtures with chemotherapeutic agents.

## 3. Photoactivators Based on Non-Porphyrin Complexes

In addition to porphyrin metal complexes, transition metal complexes, in particular iridium(III) complexes, with non-porphyrin ligands have recently been widely studied as photosensitizers for PDT due to their exceptional photobiological properties [74,75,76].

**Figure 10:**
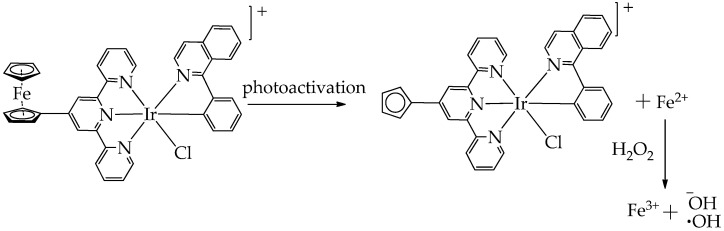
Photoactivation of complex and Fenton reaction.

Thus, in the work [77], it was established that the ferrocene–tripyridine–iridium (III) complex dissociates under the influence of light radiation with the formation of cytotoxic Fe^2+^ ions (Figure 10). Subsequently, according to the Fenton reaction, ·OH is formed, and the metal complex generates ROS, inducing ferroptosis and autophagy, which ultimately causes the immunogenic death of melanoma cells. The Ir (III) complex generated ROS when irradiated with light of a wavelength of 652 nm. As a result, the authors established that the presented iridium complex has cytotoxicity with respect to skin melanoma cells A375, as evidenced by the IC_50_ value of 1.22 ± 0.23 μM. The authors also showed that the expression levels of LC3II/LC3I (marker for autophagy), involved in the formation of autophagosomes, were significantly increased, and the expression levels of GPX4, a key endogenous inhibitor of ferroptosis that reduces the level of phospholipid hydroperoxides, were decreased after light treatment. In addition, strong green fluorescence was detected for the accumulation of lipid peroxides on the cell membrane, as well as ruptures of the outer mitochondrial membrane and the formation of autophagosomes, which also indicated the induction of ferroptosis and autophagy after light irradiation.

**Figure 11:**
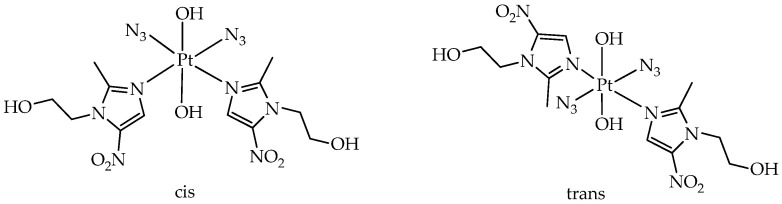
Structures of cis- and trans-complex [Pt(N_3_)_2_(OH)_2_(MNZ)_2_].

Among the platinum-group metal complexes, in addition to iridium (III), platinum (IV) complexes exhibit photoactivity. In particular, in the work [78], it was demonstrated that platinum octahedral metronidazolium complexes of the type [Pt(N_3_)_2_(OH)_2_(MNZ)_2_] (Figure 11), where MNZ = metronidazole, 1-(2-hydroxyethyl)-2-methyl-5-nitroimidazole, exhibit photoactivity and cytotoxicity towards human ovarian cancer A2780 and bladder cancer SW780 cells upon excitation by visible light and under hypoxia. Low dark cytotoxicity with IC_50_ values > 100 μM was observed for cis- and trans-isomers towards all cancer cell lines at different oxygen concentrations. It is interesting to note that trans-photoactive dimetronidazole platinum complexes have higher solubility in water, a more intense and red-shifted band, and higher phototoxicity compared to their cis-isomers. Thus, when irradiated with visible light, only the trans-isomer exhibited phototoxicity towards SW780 bladder cancer cells. For A2780 ovarian cancer cells, moderate photocytotoxicity with an IC_50_ of about 45 μM was observed for the cis-isomer only when exposed to blue light (465 nm), while for the trans-isomer, cytotoxicity was observed when irradiated with both blue and green (520 nm) light.

The authors explain the better phototoxicity of the trans-complex as follows: During photoactivation of the trans-complex, two azide radicals, N_3_· and [Pt^II^(MNZ)_2_]^2+^, are formed, which have a high affinity for biomolecules, including L-guanosine-5′-monophosphate (5′-GMP), ctDNA, and tRNA in free-cancer-cell environments. In contrast, during photoactivation of the cis-complex, one azide radical and one metronidazole ligand are formed from the trans-position, and their binding abilities are much weaker compared to those of the trans-complex.

The authors also showed that the mechanism of action of the trans-complex is to bind reactive hydroxy/aqua adducts to DNA, RNA, and proteins, as well as to induce apoptosis and damage mitochondrial membranes. Axial metronidazole conjugated to the Pt(IV) prodrug is irreversibly reduced under hypoxic conditions to retain the Pt(IV) prodrug in hypoxic areas and accumulates deep in cancer cell spheroids. Spheroids better mimic tumor biology than 2D models.

**Figure 12:**
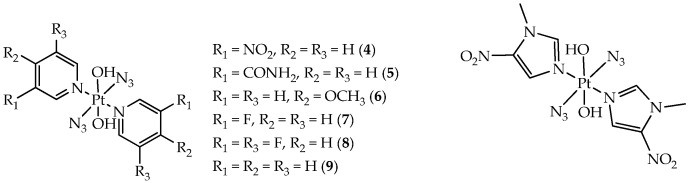
Structures of complex [Pt(N_3_)_2_(OH)_2_(L)_2_] with pyridinium and imidazolium ligands.

In the work [79], the phototoxicity of platinum (IV) complexes of the general formula trans, trans, trans-[Pt(N_3_)_2_(OH)_2_(L)_2_] with *N*-heterocyclic amine ligands (pyridinium and imidazole, Figure 12) in relation to SW780 bladder cancer cells was investigated. The introduction of *N*-heterocyclic amine ligands contributed to the improvement in the phototoxicity of Pt(IV)–diazido complexes under hypoxic conditions.

Among the pyridinium complexes, compounds with moderate electron-withdrawing and electron-donating groups (compounds **5–7**) showed low dark toxicity under normoxia and hypoxia (IC_50_ > 100 μM) against bladder cancer cells SW780. In turn, compound **9**, un-substituted, and compound 4, bearing a strong electron-withdrawing nitro group, had dark toxicities (IC_50_ 15.1–74.4 μM under normoxia, 14.4–82.2 μM under hypoxia). When irradiated with blue light, the following photocytotoxicity order was observed: **4** (IC_50_ 1.6 μM) > **8** (7.0 μM) > **9** (7.4 μM) > **7** (8.4 μM) > **6** (9.3 μM) > **5** (>100 μM) under normoxia and **4** (1.1 μM) > **8** (1.3 μM) > **9** (13.3 μM) > **7** (18.1 μM) > **6** (19.1 μM) > **5** (>100 μM) under hypoxia. Thus, complexes with electron-withdrawing substituents **4** and **8** demonstrate higher photocytotoxicity compared to complex **6**, bearing an electron-donating methoxy group, and the un-substituted complex **9**, under both normoxia and under hypoxia.

The nitroimidazolium complex was the most promising for further study, as it demonstrated low dark cytotoxicity and high photocytotoxicity upon irradiation with blue light (463 nm) with IC_50_ values < 5 μM against SW780 both under normoxia and hypoxia. Low cytotoxicity of the nitroimidazolium complex (IC_50_ 14.4 μM) was observed towards normal bladder epithelial cells SV-HUC-1 and normal lung fibroblasts MRC-5 (8.9 μM), even under irradiation, indicating its selectivity (3.3× for SW780 vs. SV-HUC-1 under normoxia).

Along with platinum complexes, ruthenium(II) complexes have demonstrated antitumor activity. In particular, the ruthenium(II)–thiophene–polypyridyl complex TLD-1433 ([Ru(II) (4,4′-dimethyl-2,2′-bipyridine)2(2-[2′,2″:5″,2″-terthiophene]-imidazo [4,5-f][1,10]phenanthroline)]^2+^), described by McFarland as an effective antitumor agent [80,81], has successfully completed phase I clinical trials for the treatment of bladder cancer [82].

**Figure 13:**
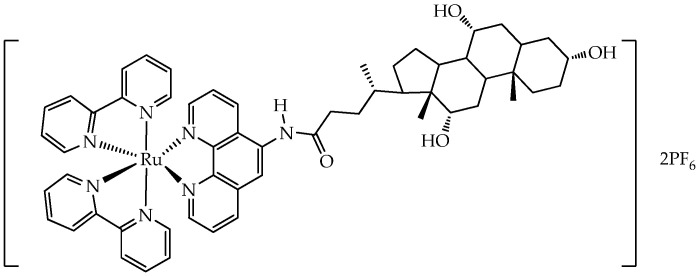
Structure of ruthenium(II) complex with cholic acid.

In turn, the ruthenium(II) complex with cholic acid (Figure 13) showed high phototoxicity (wavelength 450 nm) to human MDA-MB-231 breast cancer cells and mouse 4T1 breast cancer cells with an IC_50_ value of 1.2 μM (phototoxicity index > 83.3) and 1.7 μM (phototoxicity index > 58.8), respectively [83]. After laser irradiation, the complex generated ROS, induced ER stress, activated the STING signaling pathway in the endoplasmic reticulum (ER), and then induced the Golgi response. The stimulation led to pyroptosis and sequential activation of downstream proteins p-TBK1 and p-IRF3 of the STING pathway, resulting in the production of antitumor factors. In addition, in vivo experiments showed that the ruthenium complex has antitumor effects in the tumor model 4T1 xenograft in Balb/c mice. Flow cytometric analysis revealed an increase in the CD80/CD86 dendritic cell ratio and the appearance of toxic T cells (CD8) and helper T cells (CD4) in the tumor tissue, indicating the ability of the ruthenium complex to activate the adaptive immune response.

**Figure 14:**
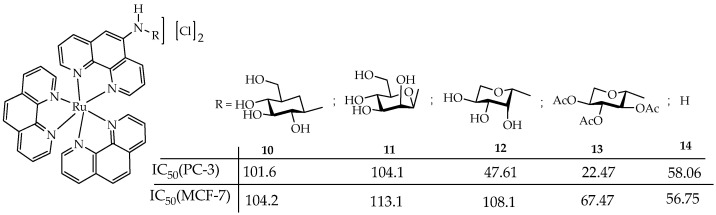
Structures of ruthenium(II) complex with carbohydrates **10–14** and phototoxicity.

In addition, some ruthenium complexes are water-soluble and photostable. For example, in the work [84], a new family of water-soluble phosphorescent ruthenium(II)–polypyridyl complexes **10–13** were synthesized, containing one *N*-(1,10-phenanthrolin-5-yl)-β-glycopyranosilamine ligand and aglycone **14** (a ligand without a carbohydrate) (Figure 14).

Compounds **10–13** were well soluble in water, methanol, and DMSO, partially soluble in acetonitrile and chloroform, and insoluble in apolar solvents such as acetone or toluene. The solubility in water of compounds **10–12** was greater than 100 mM and was due to the hydroxyl groups of the carbohydrate, and it was within 40–50 mM for acetylated carbohydrate **13** and aglycone **14**.

Compound **14** exhibited the least photostability after 5 min of blue light irradiation, while photodegradation and/or increased *N*-glycosidic bond cleavage in compounds **10–13** were detected only after 6 h of light exposure. Furthermore, compounds **10–14** lacked dark toxicity (IC_50_ > 1000 μM) but showed photocytotoxicity toward PC-3 prostate and MCF-7 breast cancer cells with IC_50_ values in the range of 48–113 μM. Blue light exposure reduced migration and increased resistance to detachment of PC-3 cells more effectively when cells were pretreated with complexes **10–14** compared to untreated irradiated cells. However, despite the better photostability of compounds **11–13**, they showed lower values of quantum yields of ROS in acetonitrile (11–37%) compared to compound **14** (53%). Only compound **10** showed a quantum yield value comparable to that of **14** (55%).

**Figure 15:**
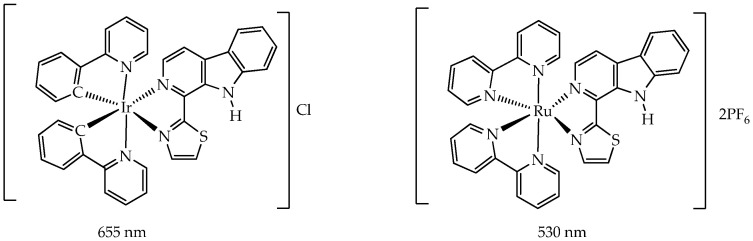
Structures of iridium(III) and ruthenium(II) complexes and λ_abs_.

In our opinion, the disadvantage of platinum and ruthenium complexes compared to iridium complexes as PSs is their photoactivation at wavelengths of less than 650 nm. In turn, it is known that red light is usually used in PDT due to the deepest penetration through tissues and the lowest absorption by physiological chromophores in cells [85]. In the work [86], it was shown that the iridium complex with the thiazolyl-β-carboline *N*,*N*-ligand (Figure 15) is activated not only by green (530 nm) but also by red (655 nm) light, while the ruthenium complex is activated only by green light. The Ir(III) complex exhibited higher cytotoxicity (IC_50_ 2.08 μM) compared to the Ru(II) complex (IC_50_ 37.27 μM) towards human prostate cancer cells PC-3. Microscopic studies showed that the Ir(III) complex rapidly penetrates cancer cells by diffusing through the cell membrane independently of the endocytic pathway, accumulates in mitochondria and lysosomes, and binds to mitochondrial and endolysosomal cellular compartments. Photoactivation of the iridium complex triggers ROS generation, disrupts mitochondrial and lysosome function, and leads to cancer cell apoptosis. The antitumor effect of the iridium complex was also studied using spheroids from human adenocarcinoma alveolar basal epithelial cells A549—microaggregates of cancer cells 50 to 100 μm in size that recapitulate some important features of solid tumors, such as nutrient, growth factor, and oxygen gradients, as well as intercellular and extracellular matrix interactions [87]. After irradiation, the IC_50_ value was 0.36 μM, demonstrating the strong ability of the complex to inhibit tumor growth upon photoactivation.

**Figure 16:**
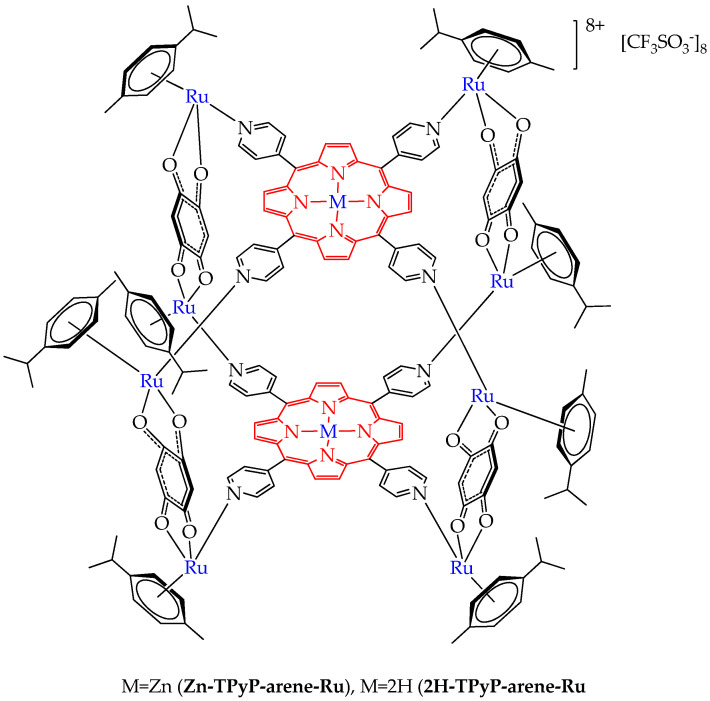
**Zn-TPyP-arene-Ru** and **2H-TPyP-arene-Ru** structures.

Non-porphyrin ruthenium metal complexes can be assembled into a single molecule with porphyrin metal complexes and porphyrins, which will allow their photoactivation with red light. Thus, the authors of the work [88] synthesized a conjugate of a ruthenium complex and zinc (II) tetrapyridylporphyrin and a zinc-free conjugate. The authors found that the zinc complex Zn-TPyP-arene-Ru had less phototoxicity (IC_50_ values 207–379 nM after 48 h) towards colorectal cancer cells HCT116 and HT-29 after irradiation with red light with a wavelength of 630–660 nm compared to the zinc-free complex 2H-TPyP-arene-Ru (Figure 16) (IC_50_ values 35–54 nM after 48 h). The study of the mechanism of cytotoxicity showed the apoptotic pathway of cancer cell death. However, the authors of this work did not provide the necessary explanation for the worse phototoxicity of the complex with zinc (II) compared to the complex without zinc (II).

**Figure 17:**
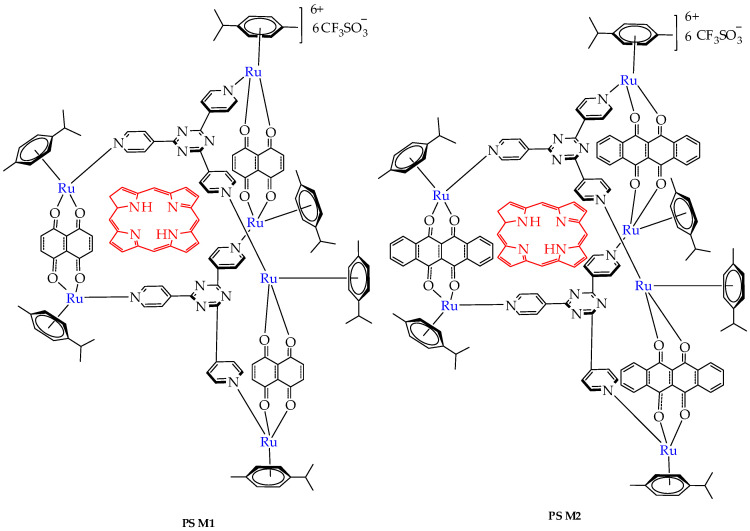
Chemical structure with the host–guest systems **PS⸦M1** and **PS⸦M2**.

**Figure 18:**
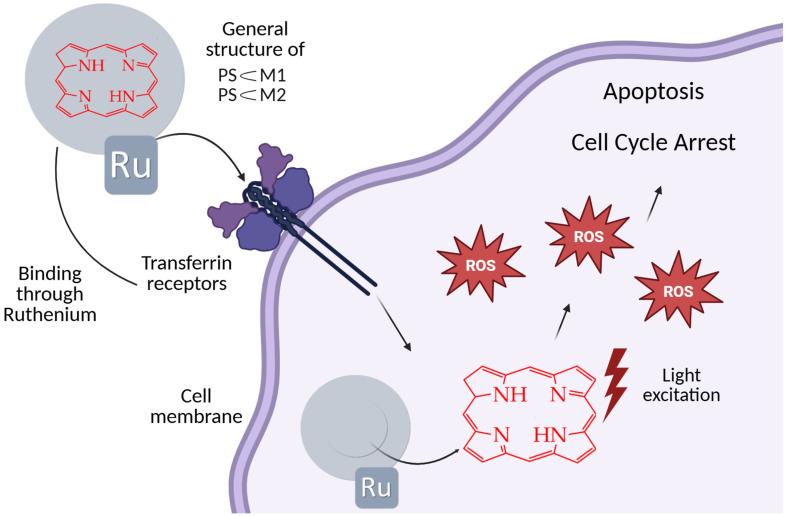
Schematic description of the cellular mechanism induced by **PS⸦M1** and **PS⸦M2**.

In the work [89], the cytotoxicity of ruthenium host–guest complexes **PS⸦M1** and **PS⸦M2** (Figure 17) containing porphyrin in the internal cavity was studied in relation to human colorectal cancer cells HCT116 and HT-29 after treatment with red light (630 nm). In this case, the ruthenium metal complex can also be considered as a porphyrin carrier, which improves its solubility in water and biological environments. The authors established the accumulation of complexes mainly in the cell cytoplasm, which potentially allows them to interact with cytosolic targets and provides significant photocytotoxicity in both cell lines after the photoactivation of the complex, inducing apoptosis through the activation of caspase-3 and cell cycle disruption (Figure 18).

In the absence of light, no significant cytotoxic effect was observed on HCT116 and HT-29 cell lines following treatment with **PS⸦M1** and **PS⸦M2**. This indicates that these compounds are non-toxic to cells in the dark, ensuring that any observed cytotoxicity can be attributed to photoactivation rather than intrinsic toxicity. Upon exposure to red light, a marked decrease in cell viability was observed in both **PS⸦M1** and **PS⸦M2** systems. The IC_50_ values for both **PS⸦M1** and **PS⸦M2** were in the nanomolar range, indicating high potency of these compounds upon light activation. For the HT-29 cell line, the IC50 values of **PS⸦M1** were 892 nM, 672 nM, and 536 nM, and those of **PS⸦M2** were 884 nM, 593 nM, and 539 nM, 12 h, 24 h, and 48 h after illumination, respectively.

Thus, among non-porphyrin complexes for use in photodynamic therapy, the most promising are iridium complexes and complexes containing porphyrin inclusions, since they absorb light in the range of wavelengths that are in the therapeutic window (600–800 nm). Nevertheless, the photophysical properties of non-porphyrin metal complexes, in particular the quantum yield of singlet oxygen, require improvement.

## 4. Photosensitizers Based on BODIPY Derivatives

In addition to porphyrin derivatives, compounds containing a 4,4-difluoro-4-bora-3a,4a-diaza-s-indacene (BODIPY) core are of interest for use as photosensitizers for photodynamic/photothermal (PDT/PTT) therapy. Unlike porphyrins, BODIPY derivatives are resistant to photobleaching and insensitive to environmental factors [90]. However, to suppress fluorescence, which is undesirable for photodynamic therapy, and to enhance intersystem crossing (where the singlet S_1_ state non-radiatively transitions to the triplet T_1_ state), as well as to impart water solubility, BODIPY must be chemically modified [91]. Despite the progress made, many BODIPYs do not reach their full potential because most of them have low ^1^O_2_ quantum yields. To overcome this problem, the incorporation of heavy atoms (such as platinum, iridium, iodine, and bromine [92]) into BODIPY structures has been widely used to increase the quantum yield of singlet oxygen ^1^O_2_ [93,94,95].

**Figure 19:**
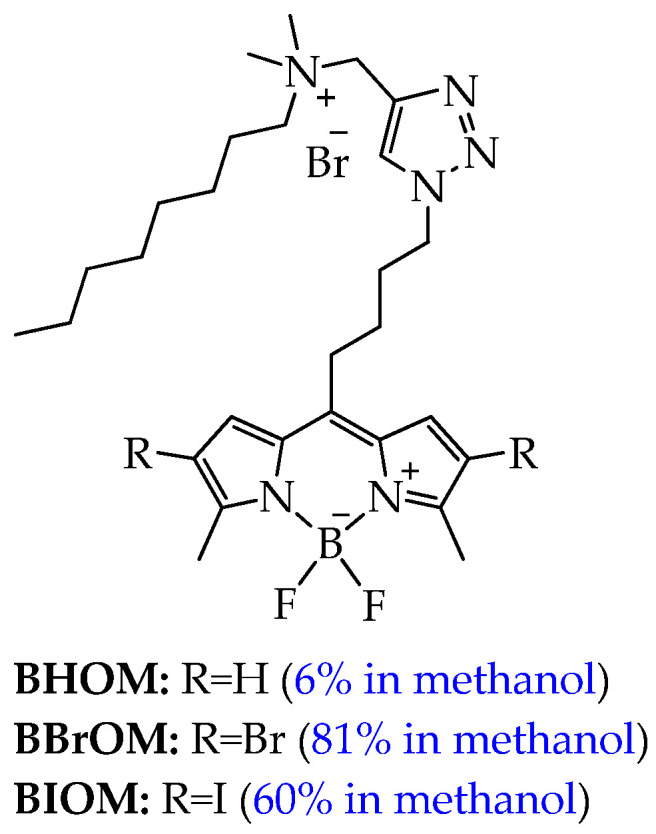
BHOM, BBrOM, and BIOM structures.

Among the BODIPY derivatives, amphiphilic BODIPY derivatives with heavy atoms can be used in photodynamic cancer therapy. For example, in the work [96], a series of three amphiphilic, mitochondria-targeted cationic boron-dipyrromethine photosensitizers of BODIPY (**BHOM**, **BBrOM**, **BIOM**, Figure 19) was synthesized.

It should be noted that **BBrOM** and **BIOM** demonstrated higher values of singlet oxygen yields (81% and 60% in methanol, respectively) compared to **BHOM** (6%) and absorbed light at a higher wavelength (red shift, for **BBrOM** from 496 nm to 520 nm, for **BIOM** from 496 nm to 527 nm). The authors explain the greater red shift for **BIOM**, firstly, by the greater polarizability and electron perturbations introduced by the iodine atom, which affect the π-conjugated system of the BODIPY nucleus and, secondly, by the strengthening of the spin–orbit coupling [97,98,99].

Conjugation of the BODIPY backbone with a positively charged lipophilic octylammonium group facilitated mitochondrial localization, which the authors confirmed by cellular uptake studies. Furthermore, the dark toxicity and phototoxicity of these cationic BODIPY photosensitizers were evaluated against two cancer cell lines (breast cancer MCF-7 and ovarian cancer HeLa). The compounds **BHOM**, **BBrOM**, and **BIOM** exhibited minimal dark toxicity against MCF-7 and HeLa cells, with cell viability remaining above 95% even at the highest concentration tested of 800 nM. **BIOM**, unlike **BHOM**, which was not phototoxic, showed toxic effects after irradiation with 515 nm light in MCF-7 cells even at the lowest concentration of 50 μM, with cytotoxicity progressively increasing with higher concentrations. A similar trend was observed in HeLa cells. In contrast, **BBrOM** showed limited cytotoxicity in both cell lines. Cell viability remained at 67%, which is insufficient for effective use in PDT. The authors explain this difference by the fact that iodine is a heavier atom than bromine, which significantly enhances the intersystem transition from the singlet to the triplet state, thereby increasing the production of ROS, such as singlet oxygen (^1^O_2_).

However, despite its higher cytotoxicity, **BIOM** induced phototoxicity in RAW264.7 murine macrophage cells at low concentrations (50 nM), while **BBrOM** and **BHOM** showed milder effects. Thus, **BBrOM** and **BIOM** are promising compounds for use in photodynamic therapy of breast and ovarian cancer. However, additional studies are required to determine their selectivity for cancer cells compared to normal cells (human mammary epithelial cells and fibroblasts).

In turn, concerns about the potential dark toxicity of heavy atoms and strong photothermal radiation causing acute pain significantly reduce patient compliance and hinder the wider clinical application of PDT using BODIPY derivatives with heavy atoms.

**Figure 20:**
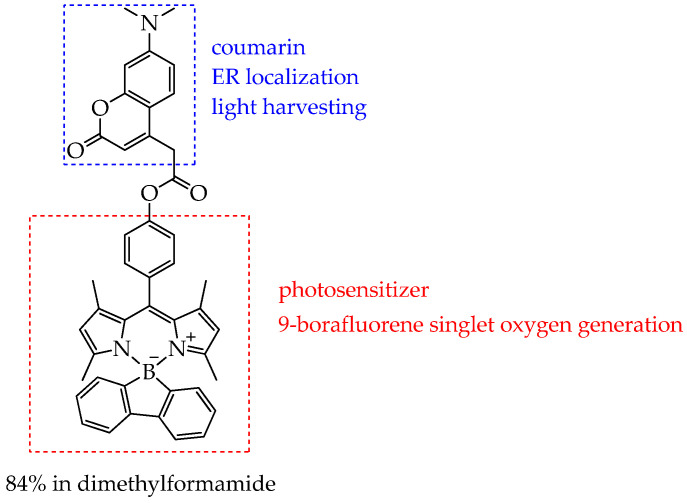
COU-BPD-BF structure.

Another approach to increasing the singlet oxygen yield, besides introducing heavy atoms into the BODIPY structure, is modifying the fluorine atoms of the BF_2_ group with functional groups that enhance intersystem crossing to the triplet state. In particular, in the work [100], fluorene was used as such a group. The authors of this work developed a new BODIPY photosensitizer free of heavy atoms (**COU-BPD-BF**, Figure 20), which has a high quantum yield of singlet oxygen (84% in DMF) due to the presence of 9-borofluorene, and it selectively localizes in the ER owing to a pendant coumarin group in the *meso* position. In addition, coumarin effectively transfers energy to the BODIPY subunit due to the antenna effect, which also contributes to the enhancement of the intersystem transition.

The selective localization of **COU-BPD-BF** to the ER promotes programmed cell death, making **COU-BPD-BF** a particularly valuable photosensitizer for PDT. However, the mechanism of action of **COU-BPD-BF** on cancer cells was not studied in this study. It was only shown that **COU-BPD-BF** was not toxic to NIH-3T3 mouse embryonic fibroblasts in the absence of light exposure. In turn, exposure to **COU-BPD-BF** and a laser led to membrane blebbing and changes in the overall morphology of NIH-3T3 cells 10 min after treatment, indicating their necrosis.

In addition, the **COU-BPD-BF** compound absorbed light at a wavelength of 488 nm (blue light). Future studies will focus on extending this approach to develop BODIPY PSs which absorb in the red region of the spectrum. In addition, further studies on the phototoxicity and selectivity of **COU-BPD-BF** are needed.

**Figure 21:**
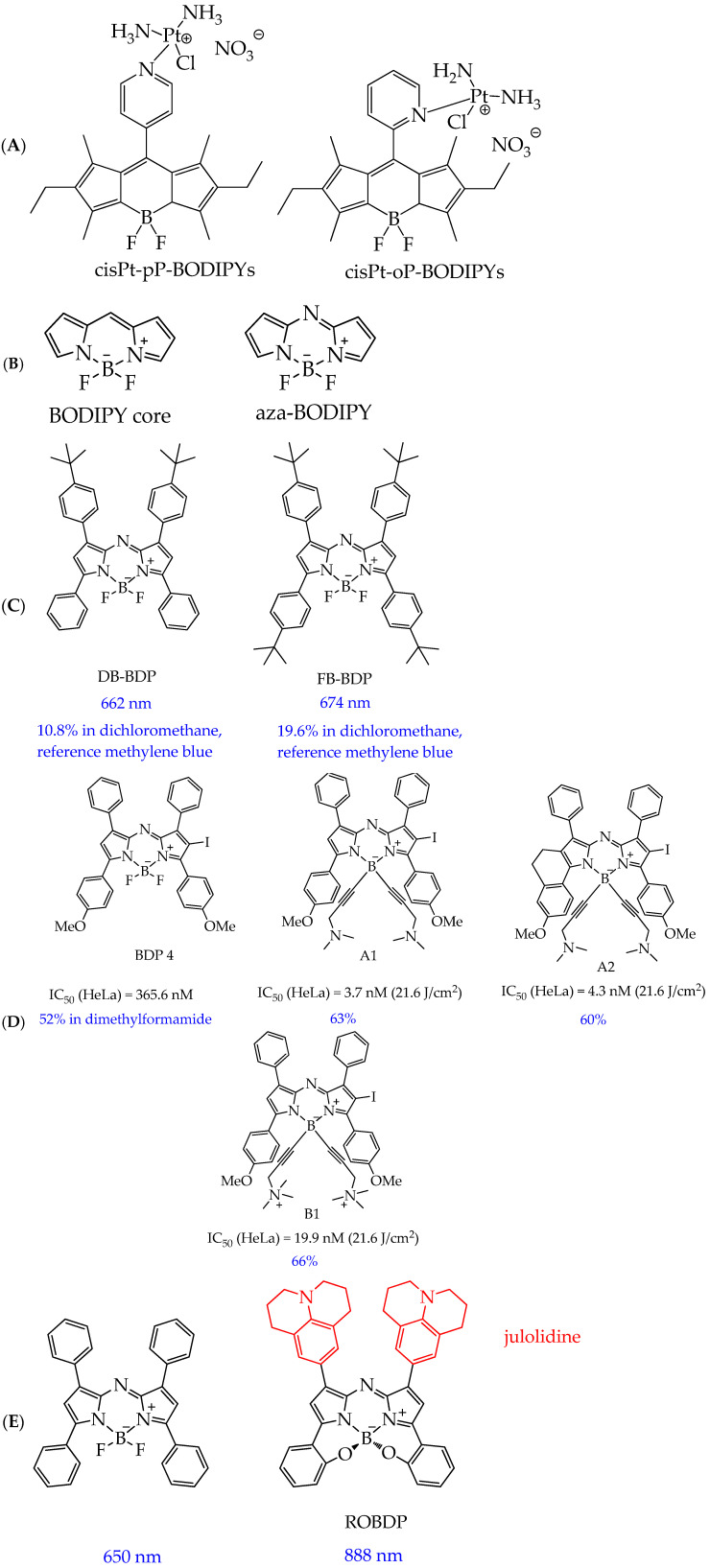
BODIPY (**A**,**B**) and aza-BODIPY (**C**–**E**) structures.

In addition to the conjugation of BODIPY with coumarin fragments, the conjugation of the BODIPY core with chemotherapeutic agents is of interest from both fundamental and applied perspectives. In particular, Ksenofontova K. V. et al. [101] synthesized conjugates of BODIPY with cisplatin, cisPt-pP-BODIPYs and cisPt-oP-BODIPYs (Figure 21A). Conjugation of the BODIPY core with a platinum-containing component resulted in a bathochromic shift (by 4–19 nm) of the absorption bands due to the heavy-atom effect. Moreover, such chemical modification resulted in a marked decrease in, firstly, the molar absorption coefficients (by approximately 1.5–3 times) and, secondly, the fluorescence quantum yields (up to 9 times) as a result of an increase in the probability of internal conversion and intersystem crossing. The obtained compounds exhibited potent photocytotoxic activity against triple-negative human breast cancer cells HCC 1806 (IC_50_ 17.8 μM and 11.8 μM for cisPt-pP-BODIPYs and cisPt-oP-BODIPYs, respectively) and cervical cancer cells HeLa (IC_50_ 21.6 μM and 34.0 μM for cisPt-pP-BODIPYs and cisPt-oP-BODIPYs, respectively).

Another promising modification of the BODIPY core is the introduction of a nitrogen atom into the *meso* position of the 4-bora-3a,4a-diaza-s-indacene ring to form aza-BODIPYs (Figure 21B).

Like porphyrins, aza-BODIPYs can absorb light in the red region and have tunable chemical structures and good photostability [102,103]. Extended π-conjugation and multiple modification sites allow fine-tuning of the photophysical properties of aza-BODIPYs, making them ideal for the design of multifunctional phototherapeutic agents [104,105]. Encouraging non-radiative decay pathways in these molecules is a key strategy for improving photothermal performance. Structural designs incorporating bulky or rotatable groups such as *tert*-butyl substituents have been shown to facilitate intramolecular motion, thereby enhancing thermal conversion efficiency while maintaining the ability to populate the triplet excited states required for ROS generation [106].

For example, in the work [107], aza-BODIPY derivatives with multiple *tert*-butyl rotor groups (**DB-BDP** and **FB-BDP**, Figure 21B) were synthesized to promote non-radiative energy dissipation and ensure efficient light-to-heat conversion.

**FB-BDP** absorbed longer-wavelength red light (λ = 674 nm in DMSO) than its di-substituted analog **DB-BDP** (λ = 662 nm in DMSO), which is an advantage of **FB-BDP** for use in PDT. Furthermore, compared to heavy-atom-containing BODIPYs, **DB-BDP** and **FB-BDP** exhibited long triplet lifetimes (7.7 μs for **DB-BDP** and 7.3 μs for **FB-BDP** at 670 nm, while for *N,N,O,O*-boron-chelated BODIPY, it is more than 100 μs [108]). This not only ensures efficient ROS generation but also avoids the unwanted dark toxicity often associated with heavy-atom-based photosensitizers. It was also found that when **FB-BDP** was irradiated with a 690 nm laser, **DB-BDP** and **FB-BDP** exhibited excellent photothermal conversion efficiency (46% for **DB-BDP** and 53% for **FB-BDP**), which makes them promising for use in photodynamic/photothermal tumor therapy.

In addition to the efficiency of photothermal conversion, aza-BODIPY derivatives containing ethynyl fragments showed high phototoxicity towards HeLa cancer cells. In particular, a number of water-soluble aza-BODIPY derivatives modified at the boron atom (compounds **A1**, **A2**, **B1**, Figure 21C) were synthesized in the work [109]. The authors found that modification at the boron atom improves phototoxicity at a wavelength of 660 nm and a low light dose of 21.6 J/cm^2^ compared to the unmodified analogue **BDP4** (Figure 21C).

The authors explain the higher phototoxicity of compounds **A1**, **A2**, and **B1** by the steric hindrance of the ethynyl groups, which disrupt intermolecular π-π stacking in an aqueous medium and prevent aggregation. In addition, the authors conducted an in vivo study on nude mice with HeLa tumors and found that tumor growth suppression after intravenous administration of compound **A1** (2 mg/kg) and subsequent irradiation exceeds the indicator for porphyrin derivatives such as chlorin e6 (Ce6) (78% for **A1** on day 24 of observation and rapid tumor progression for Ce6).

It should also be noted that compound **B1** showed reduced cellular fluorescence intensity compared to **A1**, which is consistent with its reduced photodynamic efficiency in phototoxicity assays. According to the authors, this phenomenon is due to reduced cellular internalization of cationic PSs. The quaternary ammonium groups in **B1** interfere with cellular uptake due to electrostatic repulsion with the phospholipid bilayer.

To increase the absorption wavelengths of aza-BODIPYs to approximately 900–1700 nm (NIR II region), which facilitates their deeper penetration into tumor tissues during photodynamic therapy and enhances the non-radiative transition for thermal conversion during photothermal therapy, electron donor groups are introduced into aza-BODIPY structures. In particular, in the work [110], it was found that the introduction of julolidine as a strong electron donor segment into the 1,7/3,5 sites in the aza-BODIPY system (**ROBDP**, Figure 21D) and B,O chelation promotes a red shift with an absorption maximum of 888 nm in DMSO, close to NIR II.

**Figure 22:**
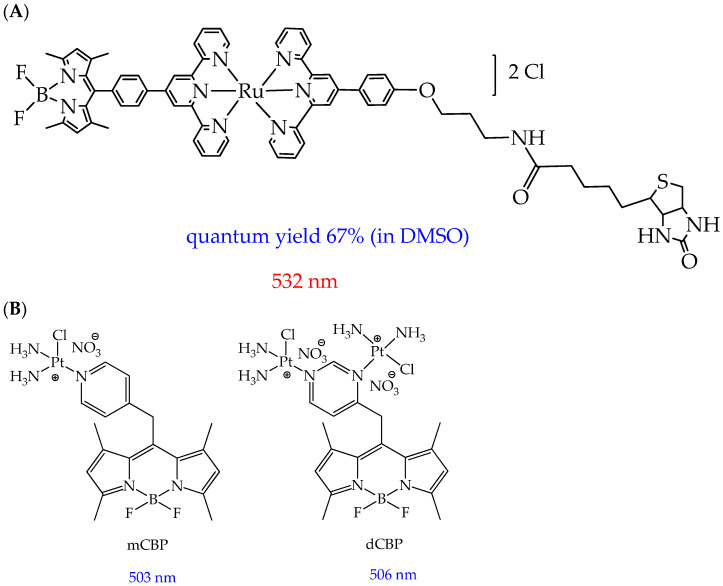
Structures of [Ru(tpy-BODIPY)(tpy-R)]Cl_2_ (**A**), mCBP, and dCBP (**B**).

In addition to molecular BODIPY, conjugates of the BODIPY core with various metal complexes can be used in photodynamic therapy. In particular, Paul et al. [111] found that a conjugate of the BODIPY core with a ruthenium(II) complex (Figure 22A) [Ru(tpy-BODIPY)(tpy-R)]Cl_2_ (tpy = 4′-phenyl-2,2′:6′,2”-terpyridine fragment) has a high quantum yield of singlet oxygen (67% in DMSO).

The authors explain this by the fact that the heavy ruthenium atom promotes spin–orbit coupling, which fills triplet states. Moreover, the obtained complex demonstrated high photocytotoxicity in human lung cancer cells A549 and ovarian cancer cells HeLa upon activation with a low light dose (532 nm, 2.2 J cm^−2^, 15 min). The IC_50_ values in relation to these cells were 0.17 μM and 0.076 μM, respectively. The photocytotoxicity of the complex exceeds the gold standard for the antitumor agent cisplatin (IC50 > 100 μM in A549 under similar conditions) by several times. The high phototoxicity of the complex against cancer cell apoptosis was due to the generation of type II singlet oxygen and a type I superoxide anion radical. In turn, photoinduced cytotoxicity toward normal human lung epithelial cells HPL1D was reduced compared to cancer cell lines. The complex was also capable of cleaving DNA in an anoxic environment, making it a photocytotoxic agent that is effective under hypoxic conditions.

In addition to ruthenium complexes, conjugates of the BODIPY core with platinum(II) complexes are also of considerable interest for research. For example, conjugates of the BODIPY core with mononuclear and binuclear platinum complexes mCBP and dCBP (Figure 22B) were shown to induce enhanced DNA damage in cancer cells due to their DNA cross-linking properties [112]. Furthermore, these compounds were shown to upregulate several key cancer-related genes (JNK (c-Jun N-terminal kinase) and Wnt/β-catenin (jnk-1, wrm-1, and gst-4)) in an in vivo study using the nematode *C. elegans* [112]. However, a disadvantage of the metal complexes considered was the absorption of light outside the therapeutic window (absorption wavelength of less than 600 nm).

Despite high singlet oxygen quantum yields and absorption maxima within the therapeutic window, BODIPY derivatives have not found application in clinical practice, unlike porphyrin derivatives. In addition, the number of publications (according to the Scopus database for the last two years) devoted to BODIPY derivatives for photodynamic therapy of cancer is much smaller compared to porphyrin derivatives (more than 600). In our opinion, this is due to issues such as unpredictable accumulation of BODIPY derivatives in tumor tissues, undesirable toxicity of some toward normal cells, as well as the unpredictability of absorption and fluorescence spectra, and the need to use complex and expensive procedures to modify the original BODIPY core.

## 5. Photosensitizers Based on Squaraines

In addition to BODIPY derivatives, a new class of photosensitizers that can be used in PDT are squaraines (SQs)—derivatives of squaric acid with a four-membered aromatic structure [113]. The possibility of using SQs in photodynamic therapy of tumors is due to the visible low-energy absorption in the red light range of the donor–acceptor–donor type associated with intramolecular charge transfer, a high molar extinction coefficient (~10^5^ M^−1^ cm^−1^), and excellent photostability [114,115]. The advantage of SQs over BODIPY derivatives is their simple structural tuning, which leads to appropriate photophysical and photochemical properties. The absorption range of SQs is modified either by changing the constituent aromatic/heteroaromatic substituents [116] or by expanding the conjugation [117,118].

**Figure 23:**
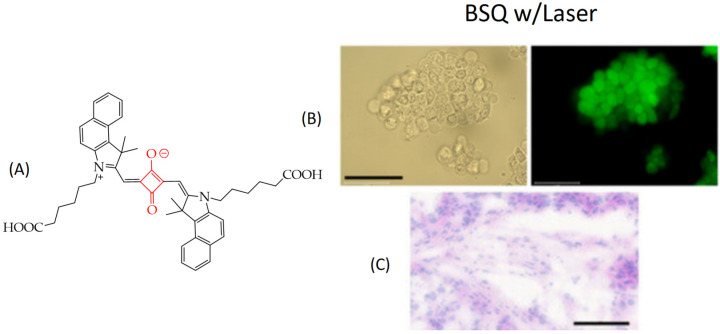
**BSQ** structure (the core squaraines (SQs) are shown in red) (**A**), fluorescence image (**B**) and histology (hematoxylin and eosin) on day 9 of observation (scale bars = 100 μm) (**C**) [119].

In particular, in the work [119], it was shown that squaraine **BSQ** modified with a benzoindole fragment (Figure 23A) demonstrated the ability to generate singlet oxygen upon laser irradiation with a wavelength of 671 nm. Efficient production of singlet oxygen was confirmed by the green fluorescent signal of human colon cancer cells HT-29 upon incubation with 10 μM **BSQ** and laser irradiation. The green fluorescent signal (Figure 23B) was associated with the transition of 2′,7′-dichlorodihydrofluorescein diacetate (DCF-DA), penetrating the cell membrane and reacting with singlet oxygen, to the DCF form.

In addition, the authors found inhibition of tumor growth of HT-29 cell xenografts in nude mice after 9 days of treatment, whereas laser irradiation alone showed no tumor growth inhibition. Histological images of tumor tissue on day 9 showed apoptotic cells, a reduced number of cancer cells, and shrunken nuclei (Figure 23C).

**Figure 24:**
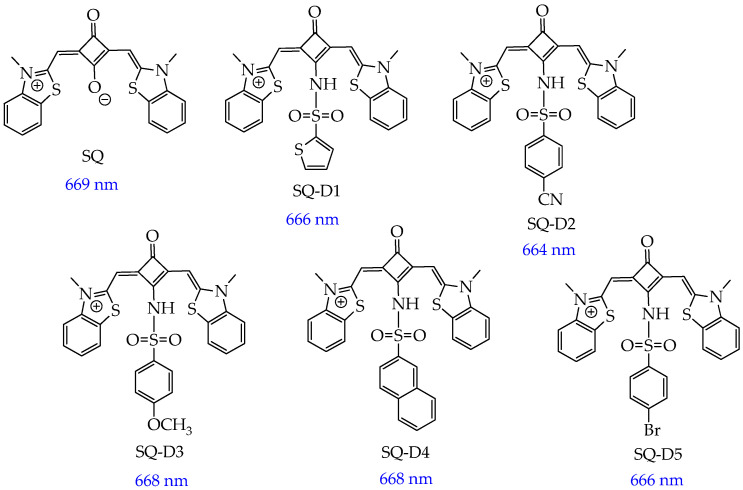
SQ, SQ-D1, SQ-D2, SQ-D3, SQ-D4, and SQ-D5 structures.

In turn, SQs containing aromatic sulfanilamide and/or dibenzothiazole fragments (SQ compounds **SQ-D1, SQ-D2, SQ-D3, SQ-D4, SQ-D5**, Figure 24) absorb red light with a wavelength of 664–669 nm [120].

The synthesized cationic SQs exhibited low dark cytotoxicity at a concentration of 100 μM toward MCF-7 breast cancer cells. After incubation of six compounds with cancer cells and irradiation (660 nm and 220 mW·cm^−2^) for 10 min, it was found that the **SQ-D2** compound containing an electron-donating cyano group in the aromatic ring exhibited the highest antitumor effect (IC_50_ = 0.25 ± 0.08 μM, cell viability was about 20% at a relatively low concentration (500 nM)). In addition, SQ-D2 reduced tumor volume in mice from ∼200 mm^3^ to 30 mm^3^ and tumor weight from 415 mg to 13 mg over 14 days of observation. TUNEL analysis of tumor tissue showed more apoptotic cells when treated with **SQ-D2** under irradiation conditions than in the dark. It was also found that **SQ-D2** did not damage the tissues surrounding the tumor and did not change the level of leukocytes, erythrocytes, hemoglobin, hematocrit, alanine aminotransferase, or creatinine in the blood of mice, which indicates the safety of **SQ-D2**.

However, the significant drawbacks of SQs are their self-aggregation in aqueous media, internal chemical instability, and physiological instability. In this regard, research in the field of SQs is mainly focused on their stabilization by nanosized delivery systems (metal–organic nanoparticles based on hexanuclear Hf6 clusters linked by benzene-1,4-dicarboxylate ligands (crystal size of about 275 nm) [121], organo-inorganic nanoparticles based on silsesquioxane [122]) as well as conjugation with β-cyclodextrins [123] or biopolymers, in particular bovine serum albumin [119] and synthetic polymers [124].

**Figure 25:**
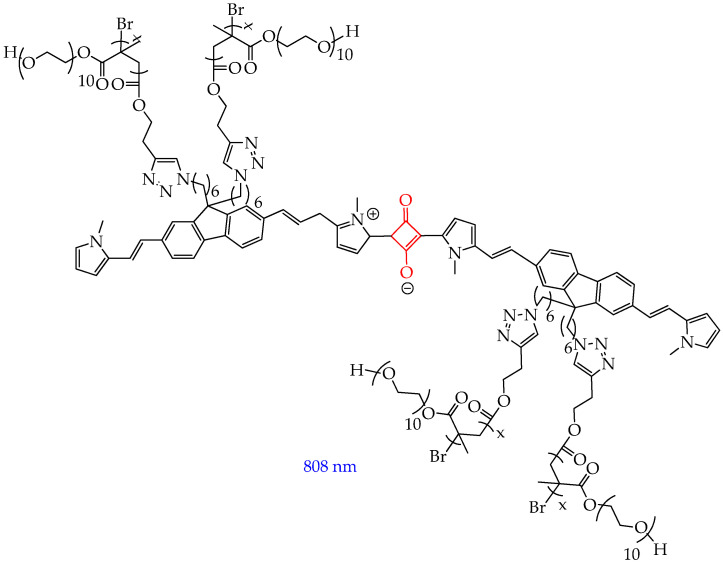
SQ-POEGMA structure.

In turn, in the work [124], a click reaction was carried out between the SQ core and the water-soluble oligomer poly(oligo(ethylene glycol) methyl ether methacrylate) (POEGMA), followed by subsequent coprecipitation with the amphiphilic copolymer PEG-b-PPG-b-PEG (Figure 25).

The obtained product was highly soluble in water, showed good stability in physiological media such as phosphate-buffered saline, Dulbecco’s modified Eagle’s Media, and fetal bovine serum. The conjugate absorbed in the NIR II region, had low fluorescence, and effectively inhibited cancer cell growth (by 94% at 12 h after injection) in mice with MCF-7 breast tumors under laser irradiation with a wavelength of 808 nm. An increase in temperature from 36 °C to 55 °C within two minutes after irradiation was also observed, indicating the photothermal effect of SQ-POEGMA.

Despite the high phototoxicity of SQs and absence of dark toxicity, the number of publications over the past two years devoted to the use of SQs in photodynamic therapy is an order of magnitude less (less than 100) than for BODIPY derivatives. In our opinion, the lower interest in SQs is not justified, since SQs have the same disadvantages as the BODIPY core (low solubility in water, the need for modification by polar fragments).

## 6. Nanosized Carriers of Photosensitizers

The hydrophobic nature of most PSs in preclinical and clinical trials limits their overall efficacy in photodynamic therapy. The use of nanocarriers in photodynamic cancer therapy is a promising direction that allows improving the bioavailability and pharmacokinetics of PSs. The accumulation of PS nanocarriers in tumor cells not only increases the local effect of PSs on molecular targets but also reduces the number of systemic undesirable side effects. The main requirements for nanocarriers have been established experimentally. Among them, the main role is played by a stable nanocarrier material subject to biodegradation by lysosomal enzymes and a minimum rate of particle aggregation in blood plasma, as well as a higher degree of phagocytosis by target cells in relation to the cells of the reticuloendothelial system.

### 6.1. Organic Nanoparticles (NPs)

Some of the representatives of nanocarriers are organic (polymeric) and inorganic NPs (nanoparticles of metals and their oxides). NPs are capable of enhancing the permeation and retention effect, transporting hydrophobic and charged PSs to the target area without degradation with a high ability to generate ROS and a minimal number of side effects [125]. The total number of articles published over the past two years on NPs for PDT according to the Scopus database is more than two thousand, which indicates a greater interest among researchers in using NPs in photodynamic therapy than PS molecules.

**Figure 26:**
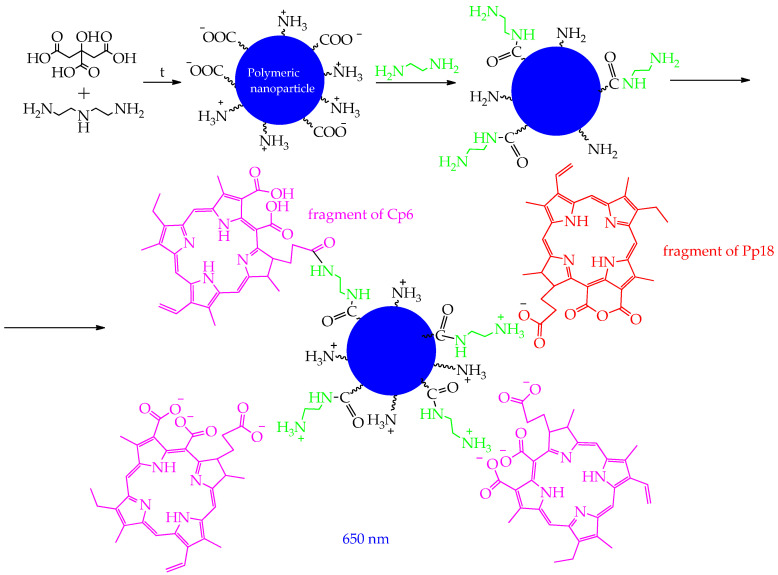
Multistep synthesis of FONPs[Cp6]. First step: synthesis of fluorescent organic nanoparticles FONPs by polycondensation of citric acid (1eq.) and diethylenetriamine (1eq.). Second step: enrichment in NH surface groups to yield FONPs^NH^_2_. Third step: preparation of FONPs[Cp6] nanoformulation by reaction with Pp18 [126].

In the work [126], by polycondensation of an equimolar mixture of citric acid and diethylenetriamine and enrichment with amino groups in ethylenediamine followed by functionalization with purpurin-18 (Pp_18_) and chlorin p6 (Cp), water-soluble and biocompatible fluorescent polyaminoamide nanoparticles FONP[Cp_6_/Pp_18_] (Figure 26) were synthesized, and the mechanism of their antitumor action under the influence of light was studied.

The NPs had a diameter of 10–15 nm and showed high solubility in water (>200 g/L) and biocompatibility. The obtained NPs had high cytotoxicity towards human colorectal cancer cells (IC_50_: 1.40 and 3.86 μg/mL for HCT116 and HT-29 cell lines, respectively) after illumination at 650 nm and insignificant cytotoxicity in HEK-293 human embryonic kidney cells at a concentration of 10 μg/mL. Subcellular localization studies showed that the NPs were distributed throughout organelles including mitochondria, lysosomes, and the ER. The authors [126] also found that NPs induced a significant increase in the rate of early and late apoptosis (up to 46.23% and 69.94% after 24 h and 48 h, respectively, for HCT116 cells and up to 28.01% and 60.19% after 24 h and 48 h, respectively, for HT-29 cells). To confirm the mechanism of apoptotic cell death observed after photoactivation with NPs, the level of caspase-3/7 activity was measured, and it was found that NPs induced a significant increase in the percentage of activated caspase-3/7 (by 86.6% compared to 6.1% for the control group after 48 h of illumination). To evaluate the late-stage apoptosis process and examine the nuclear changes induced by NPs, DNA fragmentation was measured 24 and 48 h after PDT using enzyme immunoassay. In HCT116 cells, the results showed that NPs induced a strong increase in DNA fragmentation after PDT by 5.2 and 12.3 times, at 24 and 48 h, respectively, compared to the control (hydrogen peroxide).

In addition to polyaminoamides, biodegradable synthetic and natural polymers, in particular PLGA and proteins, can be used to develop polymer NPs. For example, the authors of the work [127] used PLGA nanoparticles coated with chitosan for stabilization and grafting of folic acid to deliver hydrophobic salts of berberine (dodecyl sulfate and laurate) isolated from barberry. The average diameter of the NPs was 212 nm and 229 nm for berberine laurate and dodecyl sulfate, respectively. The chitosan shell imparts a positive charge to the NPs and improves the interaction with cell membranes, accelerating endocytosis. The positive charge also allows additional modification of the nanoparticle surface by ionic interaction with anionic molecules (in this work, with folic acid).

The authors found that the synthesized NPs effectively released berberine salts in the cytoplasm of T98G glioblastoma cells, especially in mitochondrial organelles. The NPs were effective in inducing a decrease in the short- and long-term viability of T98G cells, with berberine dodecyl sulfate-loaded NPs being the most effective due to its more efficient loading into NPs. In turn, no differences in viability were found after 24 h of observation after four-minute light stimulation in the presence of NPs for normal rat astrocyte cells. In addition, PDT (wavelength 447 nm) using NPs significantly increased early apoptosis induction events in T98G cells and led to mitochondrial depolarization, manifested in a decrease in the red/green fluorescence ratio of the membrane dye JC-1. Thus, the developed nanoparticles are effective and selective candidates for photodynamic therapy of glioblastoma.

In the work [128], it was shown that NPs of bovine serum albumin (BSA) loaded with a photosensitizer with a pyridinium positively charged cycle using non-covalent interactions (hydrophobic, van der Waals, electrostatic) in the binding pockets of BSA, under the action of a laser, can effectively switch off mitochondrial activity and subsequently disrupt tumor angiogenesis. Naphthalene rings were used to improve the absorption of red light, an *N*,*N*′-dimethylamino group was used to enhance photosensitization, and a long chain of aliphatic ether was used for hydrophobic binding to protein amino acids.

**Figure 27:**
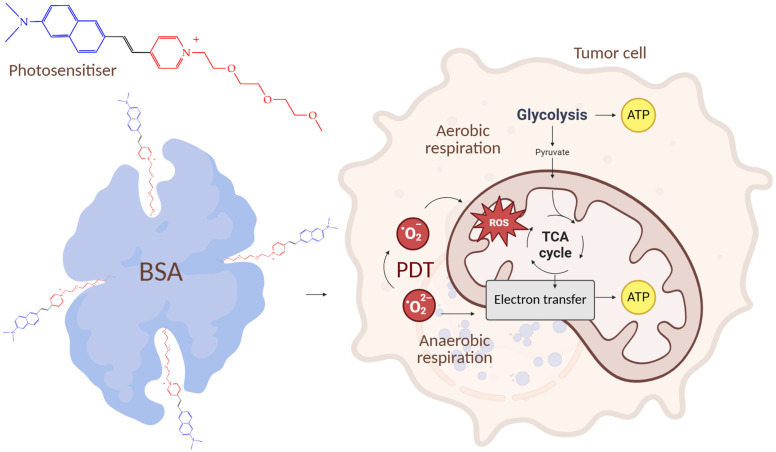
Tumor photoablation effect of the ^1^O_2_ generated by PDT results from the damage of the tumor-related vasculature leading to severe tissue hypoxia and malnutrition and to the induction of mitochondrial metabolism and oxidative stress crisis, thereby enabling precise imaging-guided tumor photoablation.

The NPs showed a high mitochondrial membrane penetration ability and induced mitochondrial morphological changes such as shrinkage, blebbing, and fragmentation. The NPs also showed a higher tumor growth inhibition rate (79.5%) in BALB/c mice bearing subcutaneous HeLa tumors compared to the photosensitizer without BSA nanoparticles (inhibition rate of 20.6%). TEM images of mitochondria showed disappearance of outer mitochondrial membranes and vacuolization after PDT. In turn, glycolysis, the glycolytic reserve, and the glycolytic capacity were significantly reduced in HeLa cells after photodynamic therapy with NPs. Figure 27 shows a general diagram of the mechanism of antitumor action of the NPs.

However, the toxicity of polymer NPs was not studied in the works considered. On the contrary, in the work of [129] toxicologic assessment of nanoparticles based on platinum porphyrin-dopeed poly (9,9-dioctylfluorerene-benzethiadiazole) having the average diameter of 18 nm, was carried out The authors showed the absence of a statistically significant change in body weight and mass (liver, spleen, lungs, kidneys) through 1, 3, 7, 14 and 30 days of observation of adult mice BALB/C after an intravenous single dose of nanoparticles (0.3 and 1 mg/kg). The study of hemolysis using the blood of the BALB/C mice has shown that nanoparticles in any of the estimated concentrations (1, 2, 5, 10, 25 and 50 mg/L). In addition, nanoparticles (1 mg/kg) did not have a negative impact on immuno-dependent hematological indicators (obstructing leukocytes, neutrophils, lymphocytes, monocytes, eosinophils, thrombinophils) in healthy mice. Biochemical analysis of the levels of alanine aminotranspharase and aspartate aminotransferase, as well as blood urea nitrogen and creatinine level in the blood serum showed the lack of significant deviations from the norm after the introduction of NPs doses of 0.3 and 1 mg/kg. Thus, nanoparticles were biocompatibility and did not induce liver and renal toxicity.

Despite the excellent antitumor properties of polymer NPs, the biocompatibility of polymers considered and accumulate in in tumor-associated macrophages [130] for a long time due to the so-called enhanced permeability and retention effect, polymer NPs have disadvantages. These include, for example, aggregation PS in polymeric nanoparticles (for PLGA NPs), which leads to a decrease in the yield of singlet oxygen [131].

### 6.2. Inorganic Nanoparticles

Among inorganic NPs, mesoporous silica nanoparticles (MSNs) can be used in photodynamic therapy due to their unique mesoporous structure, large pore volume, high specific area, good biocompatibility, and ease of surface modification [132,133].

**Figure 28:**
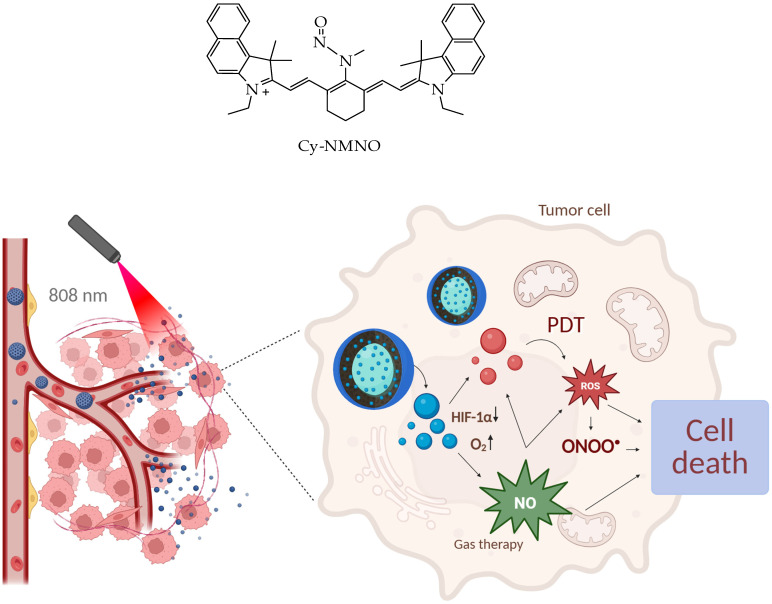
Cy-NMNO structure and mechanism of antitumor action (blue circle—Cy-NMNO; red circle—Cy (without NO group)).

In particular, in the work [134], a photosensitizer based on the fluorescent dye cyanidin Cy with the nitrosamine Cy-NMNO (Figure 28) was loaded into MSNs. In this work, the authors combined laser photodynamic therapy in the near-infrared range with gas therapy based on nitrogen monoxide, NO, which made it possible to overcome hypoxia in tumor tissues, characteristic of PDT.

The continuous consumption of oxygen to generate cell-damaging ROS during PDT further exacerbates tumor hypoxia, severely hampering the generation of effective ROS and leading to suboptimal PDT efficacy [135]. Hypoxia-induced factor 1α (HIF-1α) is a nuclear transcription factor produced by cells in response to hypoxia. It is normally located in the cytoplasm, and the α-subunit is rapidly ubiquitinated and degraded when the intracellular partial oxygen pressure is normal. However, during hypoxia, HIF-1α accumulates, translocates from the cytoplasm to the nucleus, and binds to the β-subunit, initiating a number of genes that promote cellular adaptation to hypoxia, and HIF-1α is the main hypoxia-associated gene [136,137].

The authors of the work [134] using Western blotting established a decrease in the expression of HIF-1α and an increase in the expression of apoptotic proteins Bax, caspase-9, and cytochrome C after photodynamic therapy with Cy-NMNO in human malignant melanoma A375 cells. The authors also demonstrated a synergistic antitumor effect of Cy-NMNO due to the ability of NO formed under the action of the laser to react with ROS (in particular, superoxide anion) to form more toxic active forms of nitrogen, such as peroxynitrite (ONOO-), which aggravates DNA breakage and triggers apoptosis, ultimately enhancing the therapeutic effect of ROS on cancer cells.

The optimal nanoparticle size to stimulate accumulation in the tumor site and ensure a longer circulating half-life should be in the range of 50–300 nm. Smaller NPs (less than 50 nm) have poor porosity, while larger NPs (more than 300 nm) have impaired diffusion into the tumor mesenchyme [138]. The authors of the work [139,140,141] used SiO_2_ NPs of 50–100 nm in size to conduct PDT at a wavelength of 810 nm in vivo on the A375 mouse tumor xenograft model. The authors observed a decrease in tumor volume from 200 mm^3^ to 0 mm^3^ on the 12th day after PDT with NPs.

In another study [142], MSNs were used to deliver indocyanine green (ICG) and chlorin e6 (Ce6). To incorporate ICG on the surface of the NPs, azetidine was first polymerized to form a poly(propylene imine) (PPI) hyperbranched polymer. The MSN-Ce6@PPI-ICG nanoparticles had a monodisperse spherical shape, a diameter of about 140 nm, a pore size of 3.07 nm, and a specific surface area of 829.49 m^2^/g. Photodynamic therapy using diode lasers with wavelengths of 655 and 808 nm to excite Ce6 and ICG, respectively, was applied in this work to PC3 prostate cancer cells and L929 mouse fibroblasts [142]. The authors found that after 24 h of photodynamic therapy using 100 μg/mL MSN-Ce6@PPI-ICG, the viability of PC3 cancer cells decreased by 75%. In turn, the viability of normal L929 cells after 24 h was more than 50%, which was higher compared to PC3 cancer cells (less than 50%).

The authors also observed a 33-fold higher level of lipid peroxidation, 2.5-fold and 6-fold higher levels of Bax and Bcl-2 gene expression, and a 50% decrease in caspase-3 gene expression compared to the negative control group when using an NP concentration of 100 μg/mL. The authors explain the increase in the level of lipid peroxidation by the fact that the resulting intracellular singlet oxygen attacks lipids in cancer cells and causes their peroxidation, and the increase in Bax and Bcl-2 gene expression is due to an attack of singlet oxygen on mitochondria. The activation of the Bax and Bcl-2 genes could not prevent the death of cancer cells.

Thus, the main mechanism of action of PDT with MSNs loaded with photosensitizers is photochemical interaction, leading to oxidative stress, loss of mitochondrial potential, destruction of mitochondria, and death of cancer cells.

In addition to MSNs, gold nanoparticles can be used to deliver photosensitizers to a tumor. They are biocompatible and resistant to oxidation in physiological environments in an ultradispersed state, which ensures long-term stability under physiological conditions and slow renal clearance [143,144]. Depending on the shape, size, and nature of surface functionalization, gold nanoparticles can penetrate into cells by endocytosis or be fixed on the external structures of the cell membrane [145,146,147].

**Figure 29:**
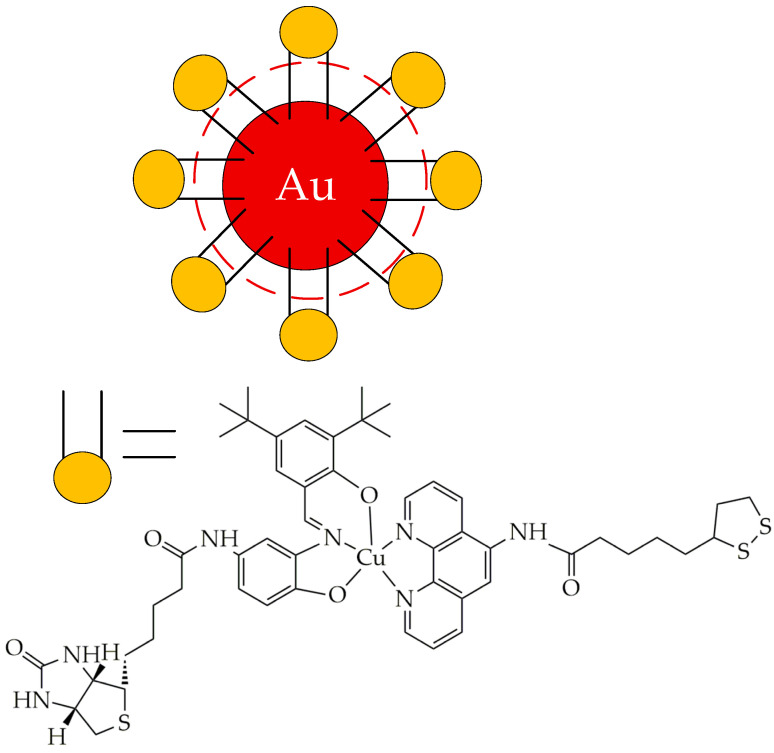
Schemes of Au NPs. λ = 600–720 nm; singlet oxygen quantum yield of 68%.

For example, in [148], gold nanoparticles were used to deliver a copper(II) metal complex [Cu(L_3_)(L_6_)], where L_3_ = *N*-(3-((*E*)-3,5-di-*tert*-butyl-2-hydroxybenzylideneamino)-4-hydroxyphenyl)-5-((3a*S*,4*S*,6a*R*)-2-oxo-hexahydro-1*H*-thieno [3,4-d]imidazol-4-yl)pentanamide and L6 = 5-(1,2-dithiolan-3-yl)-*N*-(1,10-phenanthrolin-5-yl)pentanamide (Figure 29). The resulting nanoparticles (1 mg/mL) were highly soluble in water, methanol, and ethanol and were stable at physiological pH (7.4) at ambient temperature for 3 days both in the dark and under red light. When irradiated with red light (600–720 nm), the nanoparticles demonstrated significantly higher cytotoxicity against biotin-positive human lung adenocarcinoma A549 cells (IC_50_ 13.35 μg/mL) compared to biotin-negative human embryonic kidney cells HEK293 (IC_50_ 41 μg/mL) and human lung epithelial cell line HPL1D (IC_50_ 71.5 μg/mL). The phototoxic effects of the nanoparticles are associated with the generation of singlet oxygen from molecular oxygen by reducing copper(II) to copper(I). In addition, when exposed to red light, A549 cancer cells treated with nanoparticles (12 μg/mL) showed a significant decrease (by 69%) in mitochondrial membrane potential and a significant (3-fold) increase in caspase-3/7 activity compared to A549 cells exposed to light alone. Thus, due to their phototoxicity and selectivity, gold nanoparticles can be used in photodynamic therapy of lung adenocarcinoma.

In addition to delivering photosensitizers, inorganic nanoparticles can be used to coat the core of magnetic nanoparticles, in particular, magnetite nanoparticles, which in their free form have weak absorption in the near-IR range [149]. Coating magnetite nanoparticles with metal nanoparticles makes it possible to reduce aggregation and improve dispersion of magnetic nanoparticles. In the work [150], hybrid nanoparticles Fe_3_O_4_@Au@PEG-OH and Fe_3_O_4_@Au@PEG-NH_2_ obtained by seeding spherical gold nanoparticles on cubic magnetite nanoparticles with subsequent stabilization with thiolated PEG derivatives with terminal hydroxyl and amino groups were used as photoactive agents for PDT. In turn, the polymer layer of PEG-OH and PEG-NH_2_ on the surface of the particles prolongs their circulation time and allows them to accumulate in tumor tissues. Aqueous suspensions of nanoparticles with a concentration of 1 mg/mL were irradiated with a laser with a wavelength of 808 nm for 2 min. The authors found that a suspension of Fe_3_O_4_@Au@PEG-OH and Fe_3_O_4_@Au@PEG-NH_2_ nanoparticles in water (concentration 75 μg/mL) reduces the viability of human lung carcinoma A549, diploid cell culture line composed of fibroblasts MRC-5, malignant melanoma A375, and human keratinocyte cell line HaCaT cells after irradiation with laser light (Table 2). However, PDT using nanoparticles resulted in a significant decrease in the viability of both cancer and normal cells, which is a drawback.

Despite the absorption of light by inorganic nanoparticles in the therapeutic window and the antitumor effect when exposed to a laser, there is little research interest in the use of inorganic nanoparticles (less than 100 articles in 2024–2025 according to the Scopus database). This is likely due to their poor biodegradability, which can lead to long-term accumulation in organs, potentially causing chronic damage or dysfunction. For example, MSNs are slowly biodegraded in biological environments due to the hydrolytic stability of the silica matrix. Injected gold nanoparticles can accumulate in the deep layers of the skin over time, which, combined with sun exposure, leads to gray-violet discoloration (chrysiasis) [151]. Biodegradation of 10–200 nm nanoparticles can be improved, for example, by incorporating them into a larger structure, but their elimination may still be delayed due to incomplete dispersion. Another approach is to pack the nanoparticles into polymer micelles with biodegradable functional groups (e.g., acetal) [152], as well as to modify the surface or internal structure with biodegradable polymers such as chitosan or calcium alginate [153,154].

### 6.3. Liposomes

Liposomes are multilamellar or unilamellar artificial vesicles of spherical shape, consisting of synthetic phospholipids (e.g., phosphatidylcholines) [155]. Liposomes self-assemble in an aqueous environment using hydrophobic interactions to form a sealed structure consisting of one or more lamellae having hydrophilic heads oriented toward the outer surfaces of the lamella and hydrophobic tails forming the inner part of the lamella [156]. Liposomes are designed to accumulate in tumor tissue by passive targeting (using leaky tumor blood vessels) or active targeting (using ligands on their surface to bind to specific cellular receptors) [155].

Liposomes sensitive to degradation by phospholipases had side effects and were highly systemically toxic [157]. To address these issues, attempts were made to control the release of liposomes using pH-sensitive and thermosensitive drugs [158,159]. However, the spatial and temporal settings for pH-sensitive and thermosensitive liposomes were inaccurate. In addition, thermosensitive liposomes require invasive monitoring using implantable temperature sensors and are characterized by inconsistent intratumor heating [160,161]. Therefore, the researchers decided to use photoactivated liposomes with an induced photosensitizer for precision delivery of therapeutic drugs.

Visudyne, a liposomal formulation of the hydrophobic photosensitizer verteporfin (a benzoporphyrin derivative), was approved by the FDA in 2000 for the treatment of wet age-related macular degeneration in ophthalmology [162]. Since its approval, many researchers have continued to study the use of liposomes in PDT for various cancers, particularly high-grade glioblastoma and EGFR mutations [163]. The liposomal lipid bilayer of Visudyne consists of unsaturated egg phosphatidylglycerol with a phase transition temperature of 7 °C and dimyristoylphosphatidylcholine with a phase transition temperature of 24 °C [164]. As a result, Visudyne liposomes are in a liquid phase rather than in a gel phase at body temperature (typically 37 °C) [165].

Under the action of a laser with a wavelength of 689 nm, the liposome is destroyed and Visudyne intercalated within the lipid bilayer is released, and verteporfin produces singlet oxygen in the presence of oxygen. Visudyne provides improved drug delivery, absorption, permeability, and retention in the tumor vasculature [166].

**Figure 30:**
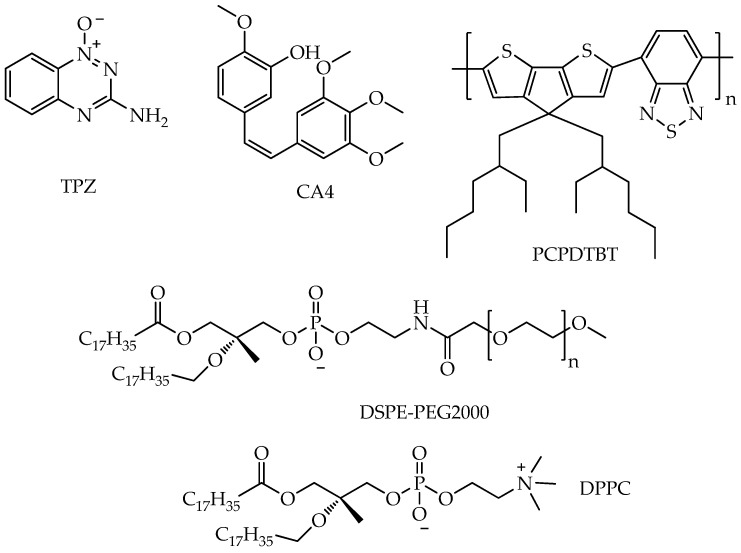
DSPE-PEG, DPPC, TPZ, CA4, and PCPDTBT structures.

In the work [167], liposomes based on 1,2-distearoyl-sn-glycero-3-phosphoethanolamine-N-[methoxy(polyethylene glycol)] (DSPE-PEG) and 1,2-dipalmitoyl-sn-glycero-3-phosphocholine (DPPC) with an average diameter of 44 nm were synthesized, containing three therapeutic agents: the prodrug tirapazamine TPZ for chemotherapy, the vascular targeting agent combretastatin A-4 (CA4) for vessel occlusion, and the semiconductor polymer PCPDTBT for PBE/ZEE; (Figure 30).

This combination therapy achieved a high antitumor effect in inhibiting tumor growth and tumor metastasis in subcutaneous mouse 4T1 breast carcinoma models. Twenty-eight hours after nanoparticle injection, mouse 4T1 tumors were irradiated with an 808 nm NIR laser, which resulted in the release of therapeutic agents from liposomes into the cytoplasm and the generation of singlet oxygen. After 10 min of laser irradiation, the tumor temperature in mice injected with liposomes reached about 60.3 °C. In turn, histological examination of the heart, spleen, and kidneys in mice after irradiation showed normal morphology, indicating biosafety of the liposomes. Absence of metastatic tumor nodules in the liver after laser treatment was also observed. The antimetastatic effect of liposomes resulted from the effective eradication of primary 4T1 tumors.

**Figure 31:**
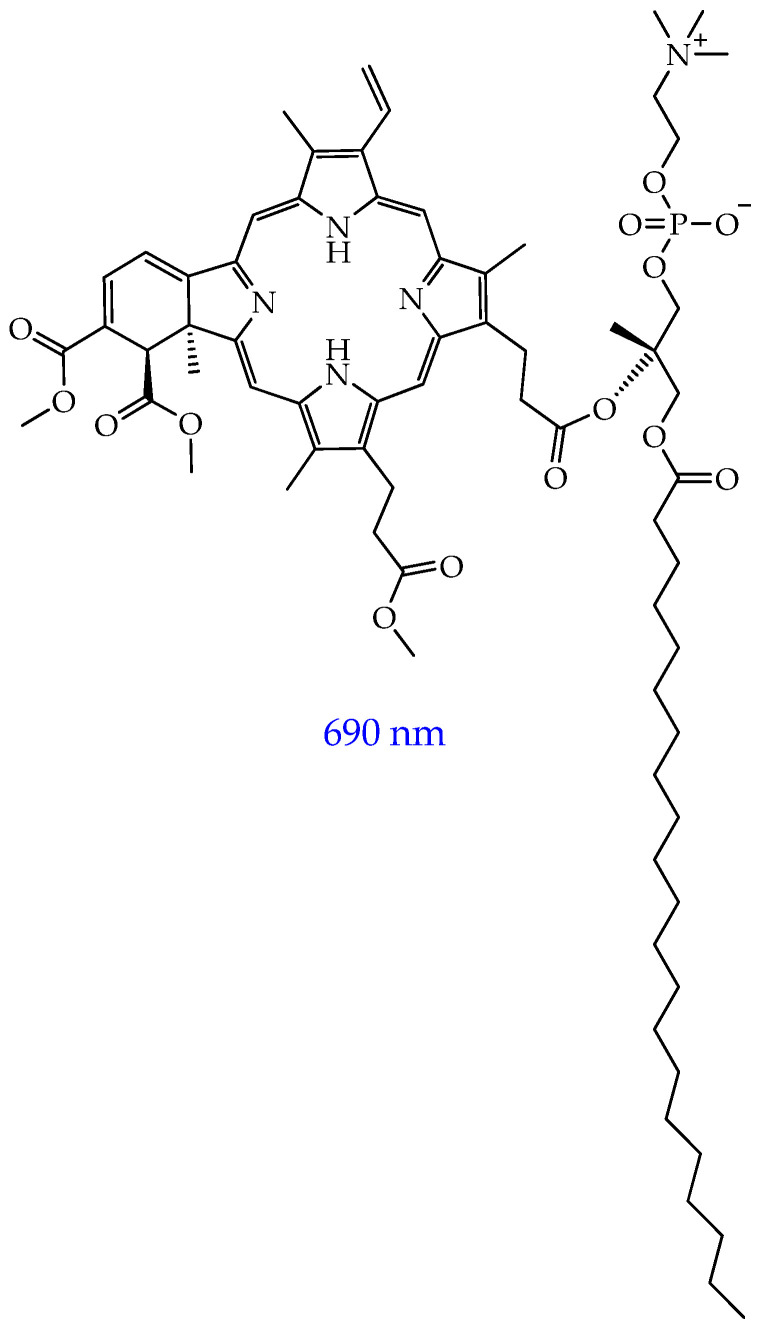
BPD-PC structure.

Photoactivatable multi-inhibitory liposomes loaded with BPD-PC (benzoporphyrin derivative conjugated to 1-arachidoyl-2-hydroxy-sn-glycero-3-phosphocholine, Figure 31) are effective treatments for pancreatic ductal adenocarcinoma (PDAC). Conjugation of α-PD-L1 antibodies to liposomes induces immunogenic cell death by blocking the PD-1/PD-L1 axis after 690 nm light irradiation and inhibits tumor growth and prolongs survival in PDAC tumor-bearing mice. Liposomes improved the median survival in mice by 26.9%, progression-free survival by 75.0%, and overall survival by 42.9% [168]. The obtained liposomes can be used in combined photodynamic therapy and immunotherapy of tumors characterized by desmoplasia—a fibrous stromal reaction leading to the deposition of extracellular matrix and promoting an immunosuppressive microenvironment.

Liposomes can be used to enhance the stability of photosensitizers. For example, indocyanine green (ICG) has several problems that hinder its clinical use, including rapid blood clearance and instability in heat, light, and solvents, which leads to the loss of photoactivation properties and PDT efficiency. The authors of the work [169] used liposomes based on 1,2-distearoyl-sn-glycero-3-phosphocholine DSPC, DSPE-mPEG5000, and methyl-β-cyclodextrin to enhance the stability of ICG and the photothermal therapeutic effect against cancer. Compared with ICG, liposomes showed a 4.8-fold decrease in degradation in phosphate-buffered saline (PBS) after 30 days. After laser treatment with a wavelength of 808 nm and an intensity of 1 W/cm^2^, the temperature of liposomes increased sharply to 63.3 °C within 10 min, indicating that liposomes converted light into heat. In in vivo experiments involving mice bearing 4T1 mammary adenocarcinoma, the tumor inhibition rate after laser treatment was 61.3%, which was approximately 10 times higher than that of ICG. In addition, histological analysis showed virtually no damage to the tissues of the liver, heart, lungs, spleen, and kidneys in mice. In turn, the levels of creatinine, blood urea nitrogen, aspartate transaminase, and alanine transaminase remained within the normal range (0.3–0.5 mg/dL, 17–38 mg/dL, 63–253 iu/L, and 35–90 iu/L, respectively) for 14 days after intravenous administration of liposomes, indicating the safety of liposomes for the kidneys and liver.

In addition to the treatment of breast adenocarcinoma, liposomes may be used to treat Human Papillomavirus-negative head and neck cancer (HNC). In [170], liposomes were used for photodynamic therapy of HNC using a chlorin-based photosensitizer, 2-(1-Hexyloxyethyl)-2-devinyl pyropheophorbide-a (HPPH). Photoactivated HPPH liposomes (660 nm, 90 mW/cm^2^, 5 min) demonstrated tumor uptake and promoted effective tumor cell killing in two mouse xenograft models, P033 and P038, of chemo-radioresistant HNC. The tumor volume was significantly reduced (from about 3500 mm^3^ to 1000 mm^3^) in the HPPH liposome and laser therapy group compared with the control group (laser only), and no signs of profound normal tissue damage or systemic toxicity were observed. The use of primary patient-derived xenograft models represents an important advance in translating preclinical findings into clinical outcomes compared to cell-line-derived models. The model used allows for the phenotypic heterogeneity and mutational burden of patient tumors to be taken into account [171]. However, although no damage to surrounding tissues was observed in the deep muscle underlying the tumors, future studies should carefully evaluate adjacent normal tissues for adverse effects.

The disadvantages of liposomes as delivery systems in the field of PDT/PTT tumors include the possibility of the oxidation and hydrolysis of phospholipids, which disrupts the structure of liposomes and leads to the premature release of PSs and complicates release control, as well as the ability of liposomes to be quickly absorbed by the reticuloendothelial system. To increase the residence time of liposomes in the blood, it is recommended to modify them with polymers (for example, PEG) [172].

### 6.4. Extracellular Vesicles

Extracellular vesicles (EVs) are natural nanocarriers produced by cells and have become a central element of drug delivery research. We classify extracellular vesicles with potential applications in the field of PDT into two main types: microvesicles and exosomes. Microvesicles are small vesicles with a diameter of 100 to 1000 nm that are released from the cell membrane following cell activation, injury, or apoptosis. Exosomes are released by exocytosis after intracellular multivesicular bodies fuse with the plasma membrane, exhibiting a diameter of 20–100 nm or 30–150 nm [173,174,175,176].

#### 6.4.1. Microvesicles

**Figure 32:**
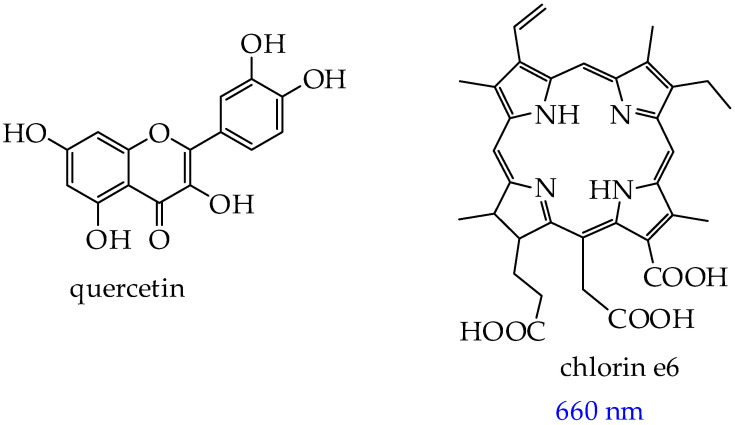
Quercetin and chlorin e6 structures.

**Figure 33:**
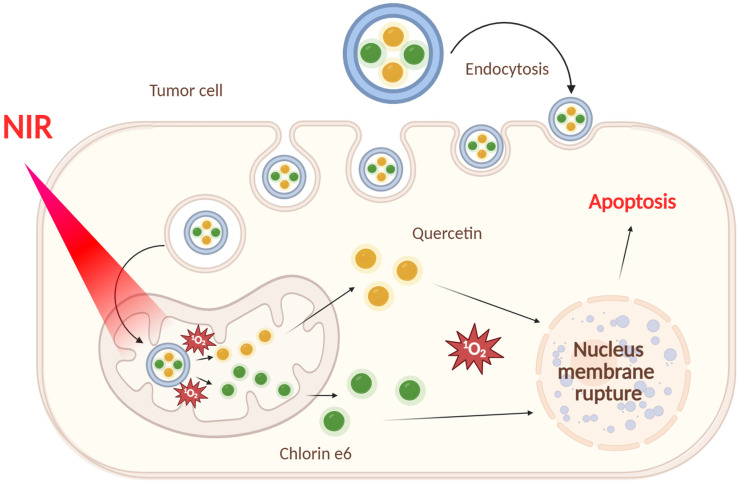
Schematic illustration of laser-induced microvesicle disruption and associated cytoplasmic transport of quercetin.

Quercetin, one of the most abundant dietary flavonoids, has potent anticancer effects. However, its application in pharmaceutical applications is hampered by poor water solubility, instability under physiological conditions, and low bioavailability. To overcome these obstacles, the authors of the work [177] proposed to use ellipsoidal-morphology microvesicles with an average size of 386 nm as carriers for the co-encapsulation of quercetin with the photosensitizer Ce6 (Figure 32). This strategy exploits the intrinsic capabilities of EVs for precise drug delivery to tumors, as well as light-activated drug release, which enables the rapid release of quercetin under near-infrared light, effectively inhibiting cell proliferation and inducing apoptosis in tumor cells (Figure 33).

Analysis of the endocytosis pathway revealed that microvesicles are internalized via an energy-dependent pathway involving clathrin-mediated endocytosis and macropinocytosis. Upon irradiation, MOC2 oral squamous cell carcinoma cells incubated with microvesicles showed a significant increase in ROS, which led to the disruption of the lysosomal membrane, facilitating the release of quercetin and Ce6 into the cytoplasm.

In vivo studies showed that quercetin-loaded microvesicles demonstrated a high tumor targeting efficiency, resulting in effective and selective tumor ablation upon photoactivation in mice bearing MOC2 squamous cell carcinomas. Almost no fluorescence was detected in the tumors for 12 h after injection. The authors of the study [177] also found that the microvesicles were safe, since they did not affect liver and kidney parameters (alanine aminotransferase, aspartate aminotransferase, alkaline phosphatase, and urea) for 28 days, did not produce morphological abnormalities in histological tissue images, and did not affect the number of red blood cells, white blood cells, and platelets in the blood of mice.

However, the interest in microvesicles compared to exosomes for the delivery of photosensitizers is undeservedly low. In our opinion, this is due to their larger size compared to exosomes, which limits their ability to penetrate deep tumor tissues and makes them more susceptible to rapid clearance by the reticuloendothelial system [178].

#### 6.4.2. Exosomes

**Figure 34:**
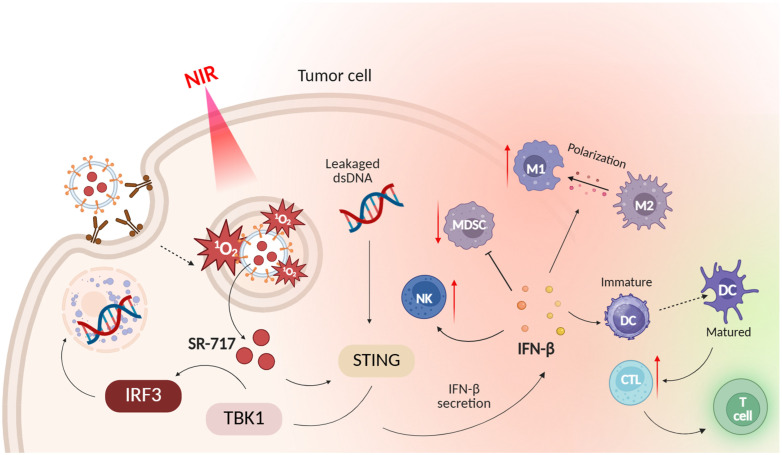
Schematic illustration of the preparation of photoactivatable tumor-cell-derived exosomes confining well-organized photosensitizer aggregates to achieve tumor-specific STING activation to safely boost synergistic photoimmunotherapy against PDAC. Once homotypic PDAC has been targeted and penetrated, the exosomes enable burst release of STING agonist and subsequent translocation from lysosomes to the cytoplasm to amplify tumor-specific STING activation, thus relieving the immunosuppression and eliciting robust antitumor immune responses to totally eradiate murine PDAC with a long-term immunological memory effect. Abbreviations: ICG, indocyanine green; ISC, intersystem crossing; ^1^O_2_, singlet oxygen; ^3^O_2_, triplet oxygen; NIR, near-infrared irradiation; cGAS, cyclic GMP-AMP synthase; TBK1, TANK-binding kinase 1; IRF3, interferon regulatory factor 3; IFN-β, interferon-β; MDSCs, myeloid-derived suppressor cells; NK, natural killer; M2 TAMs and M1 TAMs, M2 and M1 phenotype of tumor-associated macrophages, respectively; CTL, cytotoxic T lymphocyte; DC, dendritic cell.

Exosomes have been widely studied in the scientific literature from the point of view of the delivery of the photosensitizer ICG and its derivatives [179] and its co-delivery with antitumor drugs. For example, in the study [180], the photosensitizer ICG and the stimulator of interferon genes (STING) agonist SR-717 were loaded into pseudospherical exosomes (average diameter of 71 nm) obtained from 4T1 breast adenocarcinoma tumor cells. Indocyanine green formed J-aggregates in the exosome, which contributed to an increase in singlet oxygen generation from 14% for ICG to 32% for the exosome upon laser irradiation (785 nm, 0.5 W cm^−2^) due to an increase in intersystem crossing and non-radiative relaxation of excitation energy. NIR laser irradiation induced rupture of both exosomal and lysosomal membranes, resulting in photoactivated burst release and cytoplasmic trafficking of the STING agonist SR-717, which induces tumor-specific STING activation in pancreatic ductal adenocarcinoma (PDAC) (Figure 34).

The loaded exosome also demonstrated an increase in temperature of about 25.5 °C at 200 μg mL^−1^ ICG after irradiation and an increased efficiency of photothermal conversion (32.7%) compared to free ICG (15.7%). In turn, a study of the antitumor effect of exosomes containing 7.5 mg kg^−1^ ICG on mice with subcutaneous pancreatic tumors PANC02 showed rapid tumor regression with complete tumor eradication 6 days after injection and no tumor recurrence up to 28 days after injection. The immunological response revealed an increase in the levels of p-TBK1 and p-IRF3 proteins by 3.6 and 5.5 times, respectively, compared to phosphate buffer and the secretion of functional cytokines IFN-β, CXCL9, and TNF-α, stimulating the innate and adaptive immune response. Histological examination demonstrated significant necrosis and severe hemorrhagic inflammation in the tumor tissue. In addition, the exosomes did not cause harm to normal tissues of the heart, kidney, spleen, lung, and liver, indicating the safety of the exosomes.

Therefore, exosomes can be used for combined photodynamic therapy, photothermal therapy, and immunotherapy for pancreatic cancer.

In the work [181], ICG@EXOs with an average diameter of 62 nm were obtained from human oral squamous cell carcinoma (OSCC) tumor cells by ultracentrifugation at 100,000× *g* for 70 min and loaded with ICG and a chemotherapeutic drug, EGFR inhibitor, gefitinib.

As in the previous work, the authors established the J-aggregation of ICG molecules inside the exosomes. When irradiated with light of a wavelength of 785 nm, the efficiency of photothermal conversion of IG@EXOs was 27.6%, which was higher than that of free ICG@EXOs. In turn, the quantum yield of singlet oxygen was also higher (23%) compared to free ICG (14%). ICG@EXOs also showed the prolonged release of gefitinib with cumulative release rates of 62.8%, 74.1%, and 92.9% when irradiated for 2, 5, and 10 min, respectively.

Lysosomal destruction in squamous oral cell carcinoma SCC7 was confirmed by the authors using red fluorescence before irradiation and its disappearance after irradiation. A phototoxicity study showed that only 23.0% of IG@EXO-treated SCC7 cells remained alive when exposed to 785 nm light, indicating that IG@EXOs have potent photoactivated cytotoxicity. Tumor cells treated with IG@EXOs under light irradiation showed the highest level of Cl-caspase-3, a key protein in the execution phase of cellular apoptosis, compared with other control groups. Therefore, the mechanism of antitumor action of exosomes is apoptosis. In turn, IG@EXOs after light irradiation effectively suppressed tumor growth in mice with SCC7 subcutaneous tumor models with a tumor inhibition rate of 88.6%, which indicates prospects for further clinical trials of IG@EXOs.

Exosomes can be produced not only by animal cells but also by plant cells. In particular, in the work [182], exosome-like nanoparticles GDNPs@ICG were used to deliver ICG. They were extracted by ultracentrifugation from a phosphate buffer solution of ginger (*Zingiber officinale*, family Zingiberaceae) and were rich in 5-shogaol and lipids, consisting of high levels of phosphatidic acid, digalactosyl, diacylglycerol, and monogalactosyldi-acylglycerol. The GDNPs@ICG exosomes had a spherical shape and an average diameter of 124 nm. The exosomes were ruptured in a slightly acidic medium (pH 5.3) with the release of 69.4% ICG and were more stable in a neutral medium (pH 7.4) with the release of 18.1% ICG, which prevents the premature release of ICG. Under the action of a laser, GDNPs@ICG exosomes released 22.8% ICG in a neutral medium, indicating photothermal release of the photosensitizer. The photothermal action of exosomes was evidenced by an increase in the temperature of an aqueous suspension of GDNPs@ICG from 25 °C to 53.8 °C after 10 min of laser irradiation with a wavelength of 808 nm.

**Figure 35:**
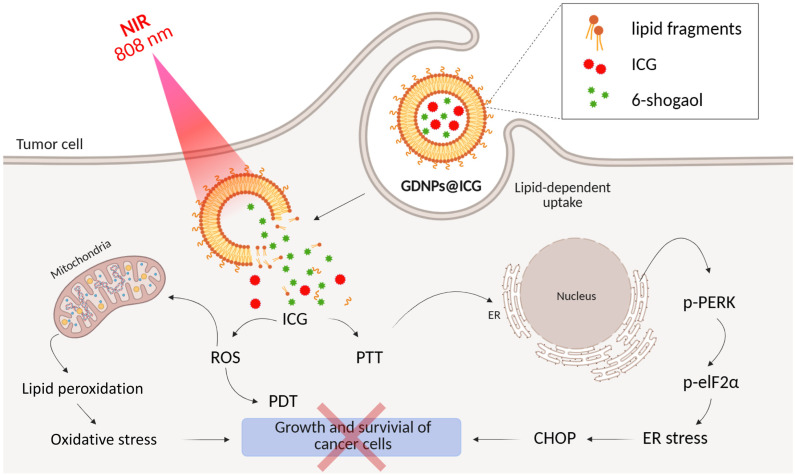
Schematic illustration of the anti-breast-tumor mechanism of GDNPs@ICG. After intravenous administration, GDNPs@ICG are delivered to the tumor microenvironment through the EPR effect and taken up by tumor cells in a lipid-dependent manner. ICG, 6-shogaol, and lipid fragments are released after lysosomal degradation or cleavage by 808 nm NIR laser. ICG, as a photosensitizer, produces a large amount of ROS and local hyperthermia, which causes lipid peroxidation and ER stress, thus inhibiting the growth and survival of breast tumor cells.

An in vitro study demonstrated the ability of GDNPs@ICG to reduce the viability of 4T1 breast cancer cells (up to approximately 40% at a concentration of GDNPs of 50 μg/mL, ICG 10 50 μg/mL, and up to approximately 5% at a concentration of GDNPs of 75 μg/mL, ICG 15 μg/mL) after laser irradiation (wavelength 808 nm), which the authors explain by the formation of singlet oxygen, lipid peroxidation, and ER stress (Figure 35). The authors confirmed this assumption by an increase in the level of the lipid peroxidation marker MDA in tumor cells and the expression of proteins associated with ER stress (β-tubulin, p-PERK, CHOP, p-elF2α).

In addition, the in vivo study showed that GDNPs@ICG significantly suppressed breast tumor growth, as evidenced by the reduction in tumor volume from about 2000 mm^3^ for phosphate buffer to about 500 mm^3^, and were biocompatible and had limited toxicity. Moreover, the authors detected reduced expression of the angiogenesis marker CD31 and N-cadherin (which promotes cancer cell invasion and metastasis), as well as increased expression of the inflammation markers IL-6, IFN-γ, and CD8 and the senescence-related genes p16, p21, and p53 in tumor tissues. The authors found that GDNPs@ICG significantly reduced angiogenesis, inhibited metastasis, activated the antitumor immune response, and promoted cell senescence in breast tumors.

In another study [183], the extract of the plant Hypericum perforatum in phosphate buffer was used to obtain HPDEN exosomes. The exosomes were loaded with the photosensitizer hepiricin (HYP). The exosomes had a two-layer cup-shaped structure with an average particle size of 67 nm. When irradiated with light of a wavelength of 590 nm with an optical power of 44 mW/cm^2^, the authors demonstrated a significant increase in the production of singlet oxygen and hydroxyl radicals from HPDENs, which was 69.98 and 2.75 times higher than in the HYP group. After 12-h co-cultivation of exosomes with human melanoma WM-266-4 cells and irradiation, a decrease in cell viability to 20% was established (HPDEN concentration of 15 mg/mL). The in vivo study also demonstrated the efficacy of HPDENs in targeting and inhibiting tumor growth without inducing organ toxicity. After 15 days of photodynamic therapy, visible tumor necrosis was observed in the HPDEN group compared to the control (phosphate buffer).

However, the general disadvantages of EVs as delivery systems compared to polymeric nanoparticles include difficulties in obtaining pure samples uncontaminated by other cellular components, blood plasma proteins, and exogenous substances; disruption of their structure during isolation using methods such as ultracentrifugation; their heterogeneity in size and origin; and their complex chemical composition [184]. These shortcomings complicate the standardization of EVs and their clinical application in photodynamic therapy.

## 7. Conclusions and Future Perspectives

Thus, key research in the field of photodynamic therapy includes the optimization of the structure of commercial photosensitizers to give them the ability to absorb light in the visible red-to-infrared spectral range and maximize the depth of light penetration, the study of delivery systems for better targeting of photosensitizers to tumor tissues, and the combination of photodynamic therapy with chemo- or immunotherapy.

Table 3 presents the advantages and disadvantages of the photoactivatable systems considered in this paper. In our opinion, the most promising photoactivatable systems for use in the photodynamic therapy of tumors from the point of view of the cost of the initial reagents for their synthesis and scale-up of production are amphiphilic porphyrins and inorganic nanoparticles (in particular, mesoporous silica particles). However, their introduction into clinical practice requires additional preclinical studies to improve their biodegradability, specifically by doping with metal ions (such as iron or sodium) or by integrating organic groups into the silica framework to weaken the Si-O-Si bonds and enhance hydrolysis.

Among the promising areas for further research is the use of temperature-sensitive “smart” nanocarriers in photothermal therapy. Such nanocarriers of photosensitizers and photoactivators may include dendrimers, liposomes, micelles, and nanoparticles based on temperature-sensitive polymers such as poly(N-isopropylacrylamide), poly(2-alkyl-2-oxazolines), and others.

It should be noted that one of the problems of photodynamic therapy is the low quantum yield of photosensitizers. In our opinion, the following methods should be used to improve the quantum yield of singlet oxygen: (1) the combined use of ultrasound and light to activate the photosensitizer; (2) modification of the structure of photosensitizers, in particular the introduction of heavy atoms (for example, bromine or iodine) into positions 2 and 6 of the BODIPY nucleus, conjugation of the BODIPY nucleus with platinum compounds, and functionalization of porphyrins at the periphery of the macrocycle with structural fragments that can be modified with carborane polyhedra; (3) the introduction of heterocyclic fragments (carbazole, pyrazine, pyrido [1,2-a]benzimidazole) into the structure of photosensitizers; and (4) the combined use of hydrophobic photosensitizers with silicon nanoparticles in photodynamic therapy. The use of special algorithms, such as the Monte Carlo method, allows the modeling of the structures of photosensitizers with the maximum quantum yield of singlet oxygen.

Among the photosensitizers with clinical applications, the second-generation photosensitizers Foscan (Germany), Purlytin (USA), Lu-tex (USA), and Photosens (Russian Federation) should be highlighted. However, despite their good efficacy in photodynamic therapy, the use of these photosensitizers has certain limitations, primarily due to the fact that a high therapeutic effect is recorded in patients with small tumors and minor invasion. Furthermore, despite their affinity for tumor tissue, they are characterized by low tumor accumulation rates and prolonged circulation in the body, which leads to significant cutaneous phototoxicity.

Among the disadvantages of the photosensitizers considered, which hinder their introduction into clinical practice, are the low solubility of some of them and the location of their absorption peaks in the region of relatively short wavelengths, which is insufficient for the destruction of extensive and deeply located tumors. To improve their solubility, the use of nanosized forms with high hydrophilicity and a positive charge [186] or the introduction of hydrophilic functional groups into their structure is required. The penetration of deep-seated tumors and the prevention of recurrence require systems that absorb in the NIR II region, a combination of photodynamic therapy with photoimmunotherapy or polychemotherapy, the development of new light delivery devices (e.g., interstitial light delivery systems), and the introduction of coherent anti-Stokes Raman scattering/four wave [187].

In turn, for the introduction of photosensitizers synthesized over the past two years into clinical practice, in addition to studying their solubility in biological environments and photophysical properties and stability in dark conditions and when exposed to light, preclinical studies are necessary: studying the kinetics of the biodistribution of photosensitizers at different doses in animal organs and tissues, studying antitumor efficacy in experimental tumor models in vivo, and assessing the dynamics of tumor growth using biokinetic analysis.

It should also be noted that the photosensitizers discussed, such as porphyrins, BODIPY, and type II-predominant squaraines, are likely to lose their effectiveness at low oxygen levels (hypoxia). Therefore, they should only be used in normoxia. In turn, type I redox systems or redox-active systems (e.g., Pt(IV)–diazido, Ir(III)–ferrocene) remain active through ferroptosis/autophagy, allowing their use not only in normoxia but also in hypoxia. Furthermore, strategies such as NO release in Cy-NMNO@MSNs or azido photochemistry in Pt(IV) complexes mitigate the limitations of hypoxia.

## Figures and Tables

**Table 2:** Viability (%) of A549 and A375 cancer cells and MRC-5 and HaCaT normal cells after PDT with nanoparticles Fe_3_O_4_@Au@PEG-OH and Fe_3_O_4_@Au@PEG-NH_2_ (concentration of 75 μg/mL).

Cells	Nanoparticles	
	Fe_3_O_4_@Au@PEG-OH	Fe_3_O_4_@Au@PEG-NH_2_
A549	59	55
A375	45	67
MRC-5	19	45
HaCaT	29	54

**Table 3:** Comparative analysis of photoactivated systems for photodynamic therapy.

Photoactivation System	Number of Research Articles in Scopus 2024–2025 and Search Query	Advantages	Disadvantages	Cost of Starting Materials for the Synthesis of a Photoactivated System (Excluding Solvents, Equipment Costs, and Other Costs) or Matrix of Nanoparticles and Cells
Porphyrin and metal porphyrin derivatives	658 (porphyrin photodynamic therapy)	Excellent phototoxicity against melanoma, lung, ovarian, and breast adenocarcinoma cells (IC_50_ less than 30 μM); selectivity against cancer cells compared to normal cells (IC_50_ more than 100 μM); and high singlet oxygen yield (more than 60%)	Absorption of light is not in the red region but in the green region for some derivatives, which leads to a low light penetration depth	Pyridine carboxaldehyde (about 200 USD/100 g), 4-acetamidobenzaldehyde (about 800 USD/100 g), pyrrole (about 80 USD/100 g), hydrochloric acid (about 6 USD/100 g), stearoyl chloride (about 260 USD/100 g), and methyl iodide (about 80 USD/100 g). Sum: 1426 USD.
Non-porphyrin metal complexes	155 (metal complex photodynamic therapy except porphyrin, porphyrins)	High phototoxicity against cells of prostate cancer, melanoma, ovarian cancer, bladder cancer, breast cancer, and colorectal cancer (IC_50_ less than 30 μM); low dark cytoxicity (IC_50_ more 100 μM); water solubility of some complexes; possibility of use not only in normoxia but also in hypoxia	Low ROS yield (less than 60%)	Ferrocene carboxaldehyde (about 1000 USD/100 g), 2-acetylpyridine (140 USD/100 g), KOH (about 150 USD/100 g), IrCl_3_·3H_2_O (17,000 USD/100 g), and phenylisoquinoline (9500 USD/100 g). Sum: 27,790 USD.
BODIPY derivatives	112 (BODIPY derivative photodynamic therapy)	Resistant to photobleaching, high singlet oxygen yield (more 60%), and phototoxicity against breast and ovarian cancer cells	Unpredictability of absorption and fluorescence spectra	2,4-dimethylpyrrole, 5200 USD/100 g; 5-bromovaleryl chloride, 1720 USD/100 g; NaN_3_, 100 USD/100 g; N-iodosuccinimide, 547 USD/100 g; sodium ascorbate, 334 USD/100 g; and CuSO_4_·5H_2_O, 114 USD/100 g. Sum: 8015 USD.
Squaraines	16 (squaraine photodynamic therapy)	Excellent photostability; simple structural tuning, which leads to appropriate photophysical and photochemical properties; low dark cytotoxicity; and phototoxicity against breast cancer cells and colon cancer cells (IC_50_ less than 30 μM)	Low solubility in water and the need for modification by hydrophilic fragments	Squaric acid, 830 USD/100 g; 1,1,2 trimethylbenz[e]indole, 480 USD/100 g; and 6-bromohexanoic acid, 267 USD/100 g. Sum: 1577 USD.
Polymeric nanoparticles	71 (polymeric nanoparticle photodynamic therapy)	Biocompatibility and biodegradation	Aggregation of PSs in some cases (e.g., PLGA) and the effect of polymer molecular weight on phototoxicity; polymers with a lower molecular weight (more than 40 kDa) form larger nanoparticles that can accumulate in tumor tissue [185]	Diethylenetriamine, 44 USD/100 g; citric acid, 2930 USD/100 g; and ethylene diamine, 45 USD/100 g. Sum: 3019 USD.
Inorganic nanoparticles	33 (inorganic nanoparticle photodynamic therapy)	Biocompatibility and ease of surface modification	Poor biodegradability, which can lead to long-term accumulation in organs, potentially causing chronic damage or dysfunction	Hexadecyl trimethyl ammonium bromide, 85 USD/100 g; NaOH, 4 USD/100 g; and tetraethyl orthosilicate, 25 USD/100 g. Sum: 114 USD.
Liposomes	223 (liposome photodynamic therapy)	Water-soluble substances can be included in the aqueous space of liposomes, and fat-soluble substances in the lipid bilayer; increased efficiency of binding to the mitochondrial membrane; membranotropism; and biocompatibility	Possibility of oxidation and hydrolysis of phospholipids, which disrupts the structure of liposomes and leads to premature release of PSs and complicates release control, as well as the ability of liposomes to be quickly absorbed by the reticuloendothelial system; high cost of synthesis	1,2-distearoyl-sn-glycero-3-phosphoethanolamine-N-[methoxy(polyethylene glycol)], 160,000 USD/100 g, and 1,2-dipalmitoyl-sn-glycero-3-phosphocholine, 80,000 USD/100 g. Sum: 240,000 USD/100 g.
Microvesicles	18 (microvesicle cancer)	Biocompatibility and in vivo safety	Limited ability to penetrate tumor tissue; difficulty of obtaining pure samples uncontaminated by other cellular components, blood plasma proteins, and exogenous substances; disruption of structure during isolation using methods such as ultracentrifugation; heterogeneity in size and origin; and complex chemical composition	Mouse oral squamous carcinoma cell lines MOC2 (about 1000–2000 USD/1 vial).
Exosomes	37 (exosome photodynamic therapy)	Natural biocompatibility, low immunogenicity, and stimulate an antitumor immune response	Difficulty of obtaining pure samples uncontaminated by other cellular components, blood plasma proteins, and exogenous substances; disruption of structure during isolation using methods such as ultracentrifugation; heterogeneity in size and origin; and complex chemical composition	Human oral squamous carcinoma cell lines SCC180, 1300 USD/1 vial.

## Data Availability

The data presented in this study are available on request from the corresponding author.

## Abbreviations

The following abbreviations and symbols are used in this manuscript:

Bax	Protein, apoptosis regulator
BODIPY	4,4-Difluoro-4-bora-3a,4a-diaza-*s*-indacene
CDBTN	13^1^-Hexamethylenediaminyl-desthiobiotinylchlorin e_6_ dimethyl ester
Ce6	Chlorin e6
DCF-DA	2′,7′-Dichlorodihydrofluorescein diacetate
DNA	Deoxyribonucleic acid
DMSO	Dimethylsulfoxide
EMA	European Medicines Agency
ER	Endoplasmic reticulum
EVs	Extracellular vesicles
FDA	Food and Drug Administration
JC-1	Dye is a commonly used tool, used for studying mitochondrial membrane potential
HIF-1α	Hypoxia-induced factor 1-alpha
ICG	Indocyanine green
MNZ	Metronidazole
MSNs	Mesoporous silica nanoparticles
NIR	Near infrared
NPs	Nanoparticles
PARP-1	Poly [ADP-ribose] polymerase 1
PEG	Polyethylene glycol
PDT	Photodynamic therapy
PLGA	Poly(lactic-*co*-glycolic) acid
POEGMA	Poly(oligo(ethylene glycol) methyl ether methacrylate)
PPI	Poly(propylene)imine
PTT	Photothermal therapy
PS	Photosensitizer
ROS	Reactive oxygen species
SQs	Squaraines
TEM	Transmission electron microscopy
^1^O_2_	Singlet oxygen
